# Highly Aligned Bacteria Cellulose Yarn Aggregation for Energy Generation and Strain Sensing

**DOI:** 10.1002/advs.202523263

**Published:** 2026-03-19

**Authors:** Chong Gao, Duo Xu, Hui Sun, Sun Ping, Zongxue Gu, Keshuai Liu, Zhiqiang Zhou, Bin Yu, Jian Fang, Weilin Xu

**Affiliations:** ^1^ College of Textile Science and Engineering Zhejiang Sci‐Tech University Hangzhou P. R. China; ^2^ Stake Key Laboratory of New Textile Materials and Advanced Processing Wuhan Textile University Wuhan P. R. China; ^3^ College of Textile and Clothing Engineering Soochow University Suzhou P. R. China

**Keywords:** bacterial cellulose yarn, core‐sheath structure, energy harvesting, strain sensing

## Abstract

Invoking high‐performance bio‐based fibres (e.g., Bacterial cellulose) contributes to the sustainability and functionality of wearable electronic devices at the material level. However, the fabrication of self‐powered and high‐mechanosensitive stretchable BC‐based sensors is challenging due to the difficulty in adaptable soft‐rigid triboelectrical interfaces and obtaining ordered conductive bacterial cellulose fibres. Here, inspired by the spiral construction from biological systems, we develop an innovative bio‐fabrication strategy to develop a core‐sheath yarn that features the ordered network and mechanosensitive twisting structures. The yarn sensor integrates the complementary advantages of triboelectric and resistive responses for the integration of strain sensing and energy self‐sufficiency. Converging factors of core‐sheath structure, modulus‐mismatch‐governed elongation, and network cracks give the yarn sensor a sensitive mechanosensitive response (8.246), a wide strain range (up to 100%), and high voltage signals (over 50 V). The scalable self‐powered fabrics based on yarns are also used as wearable power generation and energy storage for charging the yarn sensing system, achieving continuous health monitoring. The design of the unique structure assists the BC‐based sensors to effectively energy charging and driving healthy monitoring system. These empirical insights from bio‐manufacturing techniques to structural design of ordered yarns pave the way to obtaining multi‐functional high‐performance bio‐based sensors.

## Graphical abstract

Although high‐performance bacterial cellulose fibers have attracted significant interest for wearable electronics, a self‐powered and high‐mechanosensitive design is still scarce for achieving an intelligent integrated system. Here, we demonstrate an innovative bio‐fabrication strategy to develop a core‐sheath yarn that features the ordered network and mechanosensitive twisting structures to effectively energy charging and a healthy monitoring system. The proposed strategy will promote the development of fiber‐based wearable electronics with an integration of strain sensing and energy self‐sufficiency.

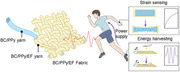

## 1 Introduction

Wearable stretchable electronics, which can be designed and produced with optimized structure and functionalities, have ushered in a new era of non‐invasive real‐time health monitoring [1, 2]. However, the drawback of wearable electronics lies on several issues [3, 4, 5]: (i) electronic waste (E‐WASTE) has become one of the major environmental pollutants, (ii) the sensing unites are generally mechanical mismatch and require improvement in their sensing performance, (iii) the weak bio‐compatible substrates impose strict restrictions on skin attachment secure and long‐term usage. In order to minimize the environmental impact on materials, bacterial cellulose (BC), which has excellent biodegradability and a unique micro‐nanonetwork structure, offers new approaches for green and bio‐friendly wearable stretchable electronics by providing a non‐toxic, natural scaffold for the sensing function [6, 7, 8, 9].

Recent research has reported that several strategies, including surface electrical modification [10, 11, 12], directional freeze‐drying [13, 14, 15], biofermentation with conductive nanomaterials [16, 17], and in situ carbonation [18, 19, 20, 21] can enable electroactive BC films. However, the prevailing and fatal issue for electronics based on BC persists is their reliance on the form of macroscopic 2D films, and the instinct rigid structure makes it difficult to balance the sensitivity and working range when worked as strain sensors. How to maximize the advantages of sensing nanonetworks and design the stretchable interface based on microarchitecture for high mechanosensitive performance has not been taken into account. In the aspect of cross‐scale structure design, ranging from BC fibers or films to yarns, that can be employed to control the inner oriented‐connected electrical networks for ultra‐sensitive stimulus response [22]. The fragmented BC films can be processed by hybridization, dopant, or polymerization as a spinning liquid, and further re‐spun for a 1D linear structure [23]. Although the reorganization structure does emerge during the spinning technologies, the electrical networks structure is not continuously and directionally reordered because the decentralized BC films are simply confined to linear space. Another aspect is that the majority of BC‐film strain sensors currently focus on mechanical stretchability from interfaced polymer substrates. Though the introduced soft‐rigid interfaces can endow crack generation and propagation of BC‐film strain sensors for great resistance variations, several performance deteriorations in these sensors, such as signal hysteresis, long response times, and poor sensing reproducibility, sometimes occur due to the internal modulus difference and stress‐distribution anisotropy from film structure [7, 8, 9]. For BC‐based strain sensors, the oriented electrical networks and localized strain concentration design for ideal sensitivity and working range are still lacking.

Except for the issues mentioned, existing BC‐based strain sensors' operation for motion monitoring requires an external power supply [24, 25, 26]. These bulky and stiff external power supplies limit their integration and practical requirements. Recently reported flexible triboelectric, thermoelectric, and photovoltaic generators have been actively evolving as environmental energy harvesters. Among them, triboelectric nanogenerator (TENG) based on BC can harvest from low‐frequency biomechanical energy and exhibit higher electrical output, but sustainability issues inevitably present. (1) Low available energy could contribute directly to powering wearable devices due to extreme resistance discrepancy from passive energy management strategies. (2) Multifunctional integrated system (strain sensing and energy harvesting) is incompatible in an all‐in‐one design, mismatch occurs between the monitoring signal range and the energy required for operation. Accordingly, a BC‐based sensing system for sustainable working should demonstrate strain sensing and energy self‐sufficiency, respectively, which is based on resistive and triboelectric responses, to provide a multifunctional design using a cross‐scale structure strategy.

**FIGURE 1:**
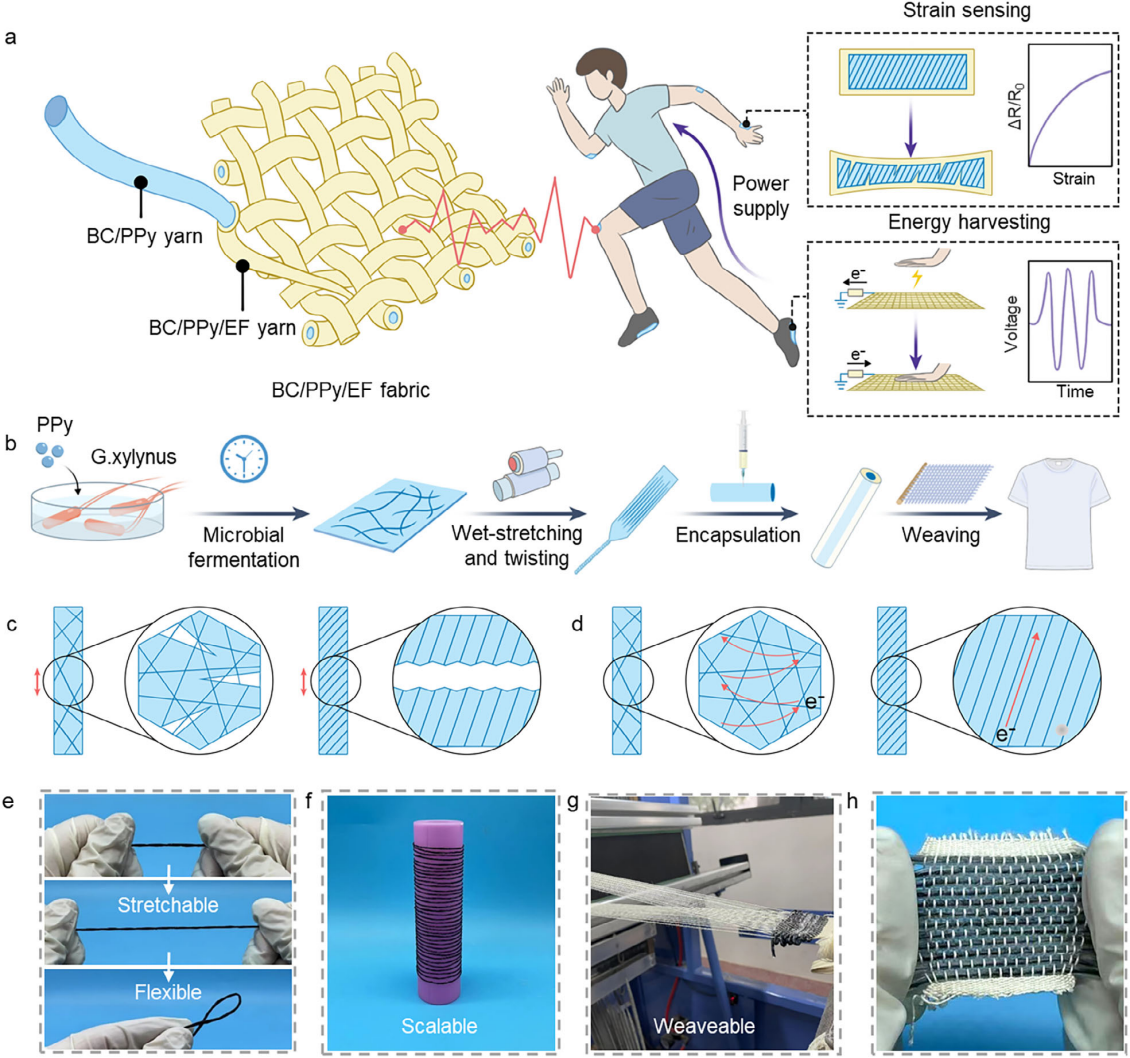
Design strategy and composition characterization of the dual‐function yarn sensor. (a) Schematic of BC/PPy/EF yarns and their aggregation for strain sensing and energy harvesting, achieving self‐produced power. (b) Schematic diagram of the fabrication process. (b) Schematic diagram showing the core‐sheath structure of BC/PPy/EF yarn. (c,d) The comparison of stretching‐stress distribution and electronic transportation behavior between the BC/PPy film (left) and yarn (right). (e–g) The stretchability, flexibility, macro‐scale preparation, and integrability characteristics of BC/PPy/EF yarn. (h) Optical image of a BC/PPy/EF yarn aggregation.

In this study, we present a novel concept of a biodegradable, designable, and scalable dual‐function core‐sheath yarn sensor in strain sensing and energy collection by combining in situ fermentation, textile technology, and polymer process in Figure 1a. The yarn sensors were fabricated by twisting in situ fermented BC/PPy composite film, and encapsulating in Ecoflex (EF) biopolymer to form the core‐sheath configuration (Figure 1b; Figure S1). The inner twisted BC/PPy layer enables a crack‐structure‐based well‐oriented conductive network with high sensitivity, and the outer Ecoflex layer endows it with excellent stretchability and durability. A stretchable yarn aggregation based on the yarns is continuously fabricated to realize energy harvesting and self‐powered sensing functions. The fabric shows good stability, washability, and high output voltage (32 V), further applied for powering the strain‐sensing system to develop a multifunctional integrated system electronics. Moreover, twisting behavior facilitates the transformation of the BC fiber network from a loose, random structure into a tight, ordered configuration, altering the mechanical and electron transport mechanisms of sensing networks (Figure 1c,d). The unique design results in excellent strain‐sensing performance at high sensitivity (GF = 8.246), wide stretching range (up to 100%), and outstanding durability (7000 cycles) for health and motion monitoring. The multifunctional yarn sensor can achieve a wide application for real‐time human‐interactive sensing systems and diverse energy‐harvesting methods, thus contributing to the further development of wearable and intelligent devices.

## 2 Results and Discussion

### 2.1 One‐Step, Scalable Fabrication of the Core‐Sheath Yarn

Bacterial cellulose (BC), as an efficient and environmentally friendly pure cellulose‐based structural and sensing scaffold featuring a unique 3D network, enables versatile tuning of material properties during secretion. In previous works, we reported an in‐situ fermentation approach for the preparation of planar BC‐based sensing films, which homogeneously incorporate conductive nanoscale blocks into the network of BC nanofibers without disrupting the continuity of the fibers [16]. Besides, this strategy achieves the spatiotemporal overlap of the critical process of BC secretion and incorporating nanomaterials [27]. Consequently, we select the in situ fermentation method to produce the conductive BC films, followed by a wet‐twisting process to construct the originated BC‐based sensing yarns with high tensile strength, processability, and wearability (Supporting Methods and Figure 1b; Figure S2). Specifically, the initial step in producing the core‐sheath structured yarns begins with microbial cultivation of bacterial fermentation products and conductive nanoparticles into the 3D electroactive network from micro to macro levels by a situ microbial fermentation process (Figure S3). Abundant active sites and high porosity on the BC promote optimal PPy deposition that achieves exceptional interfacial stability and high conductivity. Subsequently, the twisting, freeze‐drying, and encapsulation controller was applied to secure a uniform surface morphology and stable yarn formation (Figure S4a–c). The chemical components and structural confirmation of the yarn were achieved through XPS spectra, XRD patterns, and FTIR spectroscopy (Figure S5). From the optical image, it can be observed that the BC/PPy/EF yarn maintains good structural integrity, stretchability, flexibility, and continuity (Figure 1e). As shown in Figure 1f, the proposed twisting‐encapsulation technology demonstrates the feasibility of digitalized manufacturing of continuous meter‐scale BC/PPy/EF yarns. Due to the inherent properties of cost‐effectiveness, flexibility, shape adaptability, and scalability, yarns have emerged as an appealing platform for the development of flexible and stretchable sensors in massive production. Based on the interconnected conductive network and excellent stretchability, the yarn sensor with high sensitivity and board sensing range is expected to be achieved. With an industrial braiding machine, the large‐scale fabrication of yarn aggregates and plain fabrics can be achieved (Figure 1g,h). Such yarn‐based architectures permit seamless integration into body‐conforming smart apparel for continuous, constraint‐free biometric surveillance and energy harvesting in accordance with the disparity of electron affinity.

### 2.2 Structure Design and Composition Characterization

**FIGURE 2:**
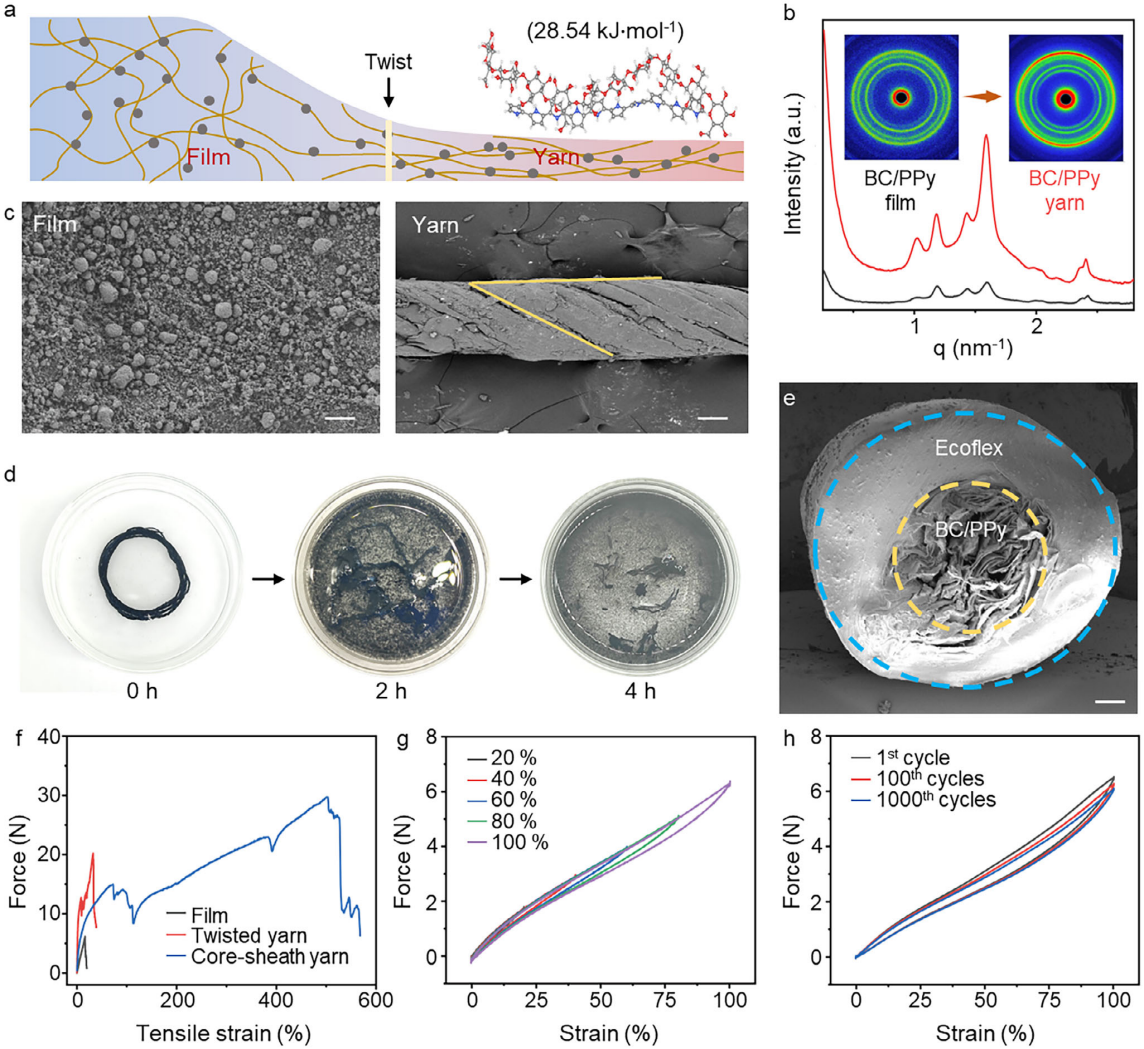
Structural characterization and mechanical properties of the yarn. (a) Schematic depicts the twisting‐induced orderliness conversion of BC/PPy fibers. (b) 1D SAXS curves and 2D SAXS patterns of BC/PPy film and yarn. (c) SEM images show the structure of BC/PPy film and yarn. Scale bar 100 µm. (d) Degradation characteristics of BC/PPy yarns within 4 h. (e) SEM images of BC/PPy/EF yarn's inclined cross‐section. Scale bar 200 µm. (f–h) Systematic comparative analysis of mechanical properties: (f) breaking strength, (g) strain gradient, and (h) stretching‐releasing cycles.

High‐oriented BC/PPy core yarns were directly counterclockwise wet‐twisted from randomly distributed BC/PPy hydrogel films grown from the culture medium (Figure 2a). The binding fastness between BC and PPy is crucial for the stability of the sensing network. Their molecular interactions were investigated by topology analysis based on the atoms‐in‐molecule theory. As shown in the insert in Figure 2a, the absolute value of the total hydrogen bond energies represented a value of 28.54 kJ·mol^−1^, the high hydrogen bond energy indicated tight intermolecular contacts for mechanical dynamic stability. The twisting behavior promotes fiber orientation, which is the dominant feature in controlling the mechanical and physical properties of fiber assemblies [28, 29, 30]. The evolution of orderliness caused by twisting was monitored by small‐angle X‐ray scattering (SAXS). The BC/PPy film exhibits a nearly homogenous diffraction ring owing to its disordered structure (Figure 2b), while the yarn yields a high intensity diffraction pattern perpendicular to the axial direction. The increase in peak intensity in the 1D integral curves further provides evidence of twist‐induced orientation. The unique twisted alignment and moderate orderliness of the BC/PPy yarn yielded the following advantages for the yarn sensor: (1) Moderate orderliness extensively disperses multidirectional loads as well as facilitates energy dissipation via the relative sliding of the hierarchical wrinkled interface and nanofibers. (2) Increased fiber orientation improves the mechanical strength and stretching stability. (3) A Shortened interlamellar interface can largely broaden the valid and accessible conductive pathway. The synergistic effects on mechanical properties and sensing performance will achieve our goals for wearable, durable smart clothes. Scanning electron microscopy (SEM) images (Figure 2c; Figure S6) confirm the planar, disordered network structure of the BC/PPy films. In the enlarged image, the PPy agglomerates, ranging in diameter from 2–5 µm, are widely distributed throughout the bacterial cellulose (BC) nanonetworks, which have a diameter of approximately 33 to 73 µm. They collectively create an ultrafine 3D nanosensing network characterized by rough surfaces and dense interiors. The twisted yarn exhibits a typical Z‐twist helical structure with a diameter of about 300 µm and a bias angle of 29.1°, showing a distinct moderate twisted alignment. Notably, the diameters of the yarn could be precisely regulated by selecting films with different thicknesses. Furthermore, the yarn demonstrates structural stability and fineness tunability, as no noticeable partial twisting or untwisting is observed in the relaxed state. Continuous cellulase treatment could cause the BC/PPy yarn to become loose, accompanied by the edge of black fibres dispersed in solution. Finally, the cellulase solution completely degraded the BC/PPy yarn within 4 h, showing excellent sustainability and degradability, showing excellent sustainability and degradability as shown in Figure 2d.

To obtain desired stretchability, the BC/PPy core yarns were encapsulated with Ecoflex (EF) to obtain BC/PPy/EF yarns, the SEM and Micro‐CT images of which exhibited a clear core‐sheath structure (Figure 2e; Figure S4c–e). The boundaries of the core and outer sheath layer are delineated by blue and yellow dashed lines, with respective diameters of 1.58 and 844 µm. Our core‐sheath structured yarn exhibits similar dimensional characteristics to commercially available yarn systems, including polyethylene rope, basalt yarn, carbon rope, and the basalt/cotton rope (Figure S7). The yarn can achieve a tough and conformal adhesion to human skin by forming a smooth and flexible interface, while the twisted core yarn wound inside the BC/PPy/EF yarn forms a continuous conductive path for sensing.

More conductive fibers were rationally contacted and arranged when applied with varied PPy concentration, twisting factors, and encapsulation diameters (Figure S8). Especially, the highly aligned BC/PPy network along the axial direction is expected to significantly reduce electrical resistance from 260.14 to 63.64 kΩ∙cm^−1^, which significantly enhances the conductive pathway and modifies the electron transfer. The tensile properties of BC/PPy/EF yarn were investigated in Figure 2f–h. The results show a remarkable enhancement of BC/PPy/EF yarn tensile capacity (518.85%) was observed relative to BC (4.53%) and BC/PPy (17.68%), and the force‐strain curve of BC/PPy/EF yarn exhibits several decentralized fractures. The difference between the elastic modulus of the core layer and outer layer progressively becomes more pronounced with increasing tensile ratio, indicating inner crack generation of BC/PPy/EF yarn from strain‐induced stiffening effects of modulus difference. This significant improvement in mechanical properties stems from the addition of Ecoflex, providing excellent tensile properties to the sensor. Furthermore, the stretch‐recovery performance of pre‐stretched BC/PPy/EF yarn is shown in Figure 2g,h, exhibiting a durable and stable recovery within a strain range of 0%–100% even after 1000 stretch‐recovery cycles. Based on the aforementioned findings, we conclude that the BC/PPy/EF yarn with twisted alignment and orderliness is essential for macroscopic mechanical reinforcement design and maintaining the electrical stability of fabricated yarns.

### 2.3 Core‐Sheath Yarn Arrays for Energy Harvesting

**FIGURE 3:**
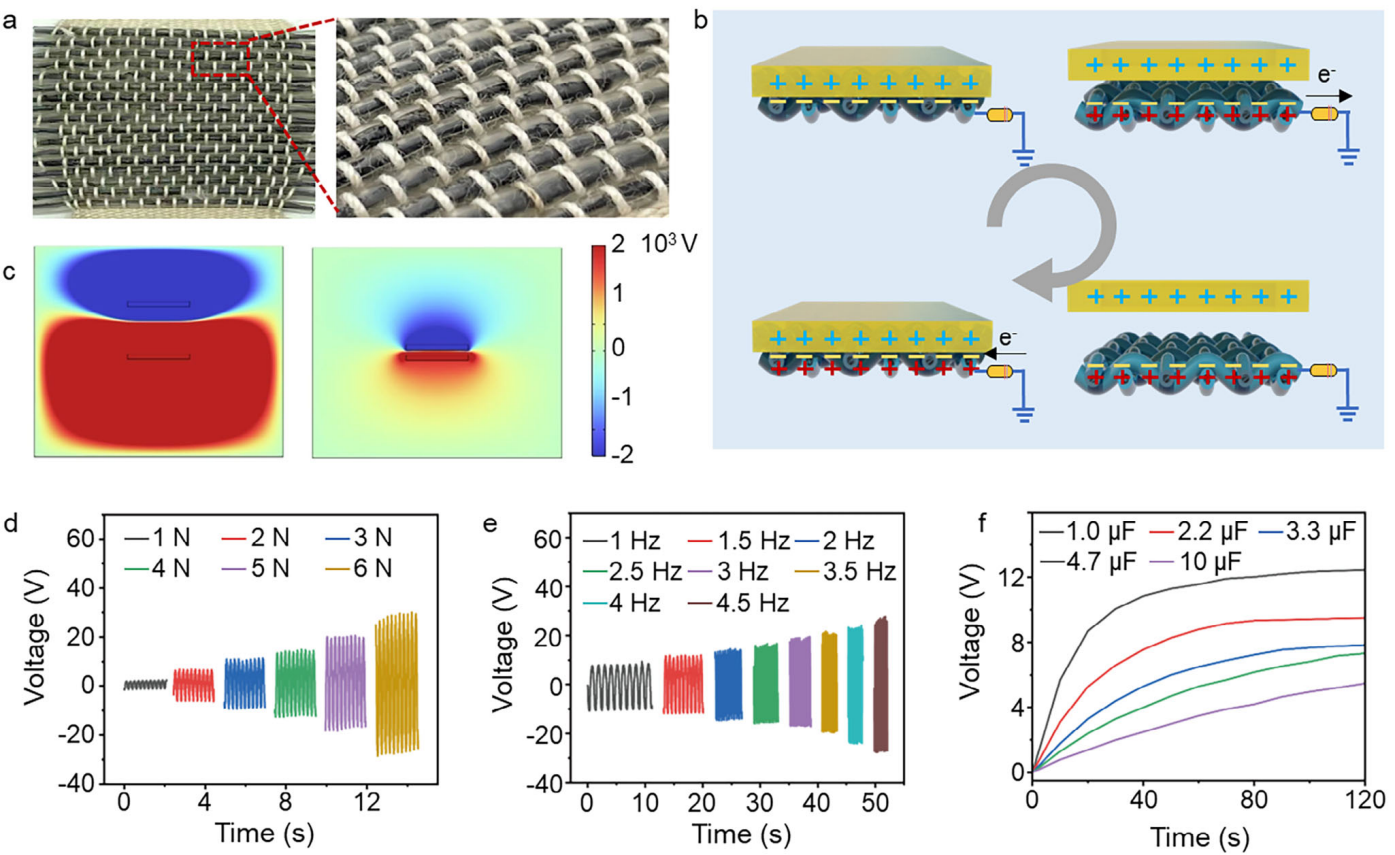
BC‐based yarn aggregation for an energy harvesting system. (a) Optical photographs of a plain fabric woven by BC‐based yarns. (b) Schematic diagram of the working principle. (c) The COMSOL Multiphysics software simulates the corresponding potential distribution of the fabric. (d–f) Charging capability of the fabric under different (d) pressure (1‐6 N), (e) striking frequencies (1–5 Hz) and (f) capacitance capacities (1–10 µF).

The core‐sheath structured BC yarns, featuring differentiated electron affinity and weavability, possess intrinsic merits as self‐powered sensing devices. By mixing‐woven with our yarns (functions as the weft‐yarns) and cotton, a scalable and flexible plain fabric is fabricated. The fabric exhibits high air permeability, conformability, and no irritation to human skin, making it suitable for clothing, insoles, and camping equipment (Figure S9). Figure 3a provides a structural visualization of the manufactured fabric, revealing the periodic unit typical of plain‐woven topologies. The yarn aggragation employs an Ecoflex outer layer with high electron affinity potential as the negative triboelectric layer for charge generation, while the inner core yarn functions as electrodes for charge collecting. When the inner yarn sensor is connected to the ground via a metal wire, the fabric operates in a single‐electrode mode. Certain materials with a propensity for electron loss—including polymethyl methacrylate (PMMA), polyimide (PI), cotton, polyethene terephthalate (PET), and aramid—can function effectively as positive triboelectric layers. Among these types, PMMA with the highest ionization energy demonstrates superior electrical output performance when contacting our yarn aggregation, rendering it the optimal candidate for the positive triboelectric layer (Figure S10a). The working principle for energy harvesting is based on the coupling effect of triboelectrification and electrostatic induction. Taking a contact‐separation movement between yarn aggregation and the PMMA plate as a relative motion cycle, Figure 3b schematically illustrates the underlying energy harvesting mechanism. This process consists of four stages: (i) Triboelectrification induced when Ecoflex contacts the palm. Ecoflex captures negative charges, while no current is through the external circuit. (ii) Inductive power in the inner electrode, when the palm is withdrawn. Free electrons flow to the ground. (iii) A new electrical equilibrium occurs when the palm is far away. (iv) Electronic reflux when the palm approaches again. Repeating this contact‐separation process can produce a continuous alternating current. The electric potential transformation of the mix‐woven fabric during contacting and separating states is simulated using COMSOL Multiphysics software to quantify the power generation process. As illustrated in Figure 3c, the evident negative potential could be observed on the surface of the fabric, which highlights the consistency of the proposed operating mechanism. It should be noted that strain‐induced bulging in self‐powered textiles during stretching leads to reduced effective contact area, consequently diminishing the voltage gradient (Figure S10b). Once the strain returns to its original configuration, the output voltage of the fabric almost completely recovers to the initial values. Exceptional stretchable and recovery properties of the cellulose‐based weft yarn enable approximately uniform output voltage maintenance over 10 000 operational cycles, indicating the potential for stable and reliable power generation in long‐term applications (Figure S10c).

The adaptability and electrical output performance of the fabric to diverse application scenarios were demonstrated by applying a series of forces (1–6 N) and frequencies (1–4.5 Hz) of external incentives. As illustrated in Figure 3d,e, the fabric sensor exhibits frequency and force dependency, with a climbing trend in the voltage signals (over 50 V) observed as both the increasing applied pressure and force frequency. The force‐dependent and frequency‐dependent characteristics of the output voltage curves prove the fabric's superior charge‐capturing ability, facilitating rapid charge accumulation. Under identical force or frequency conditions, the fabric presents consistent cyclic pulse signals, demonstrating its reliable reproducibility in sensing various external stimuli. In addition, the reason behind the self‐powered energy generation lies in the strain‐induced deformation of weft yarns. From that perspective, we realized cellulose‐based yarns have distinct gradient‐increasing voltage behaviors under varying stretching conditions when attached to fingers (at flexion angles of 30°, 60°, and 90°) or embedded in athletic socks for pedaling (at heel‐strike heights of 2, 4, and 6 cm) (Figure S11). Hence, the electricity harvested from the fabric sensor can be further stored in energy storage devices (e.g., capacitors) for subsequent usage. The effects of capacitance capacity (1.0–10 µF) on the charging ability of the fabric sensor were investigated in Figure 3f. The charging velocity of the fabric becomes slower with the increase of capacitance, and eventually reaches approximately 12 V. By charging capacitors with sufficient time and transferring to the sensing yarns, the long‐term working of the integrated low‐energy consumption system is available. As a proof‐of‐concept, the rectified bridges transformed the produced alternating current (AC) to direct current (DC) when tapping the energy harvesting fabric repeatedly, which was sufficient for lighting up the LED arrays and powering the capacitor (Figure S12).

### 2.4 Sensing Performance and Mechanism Analysis

**FIGURE 4:**
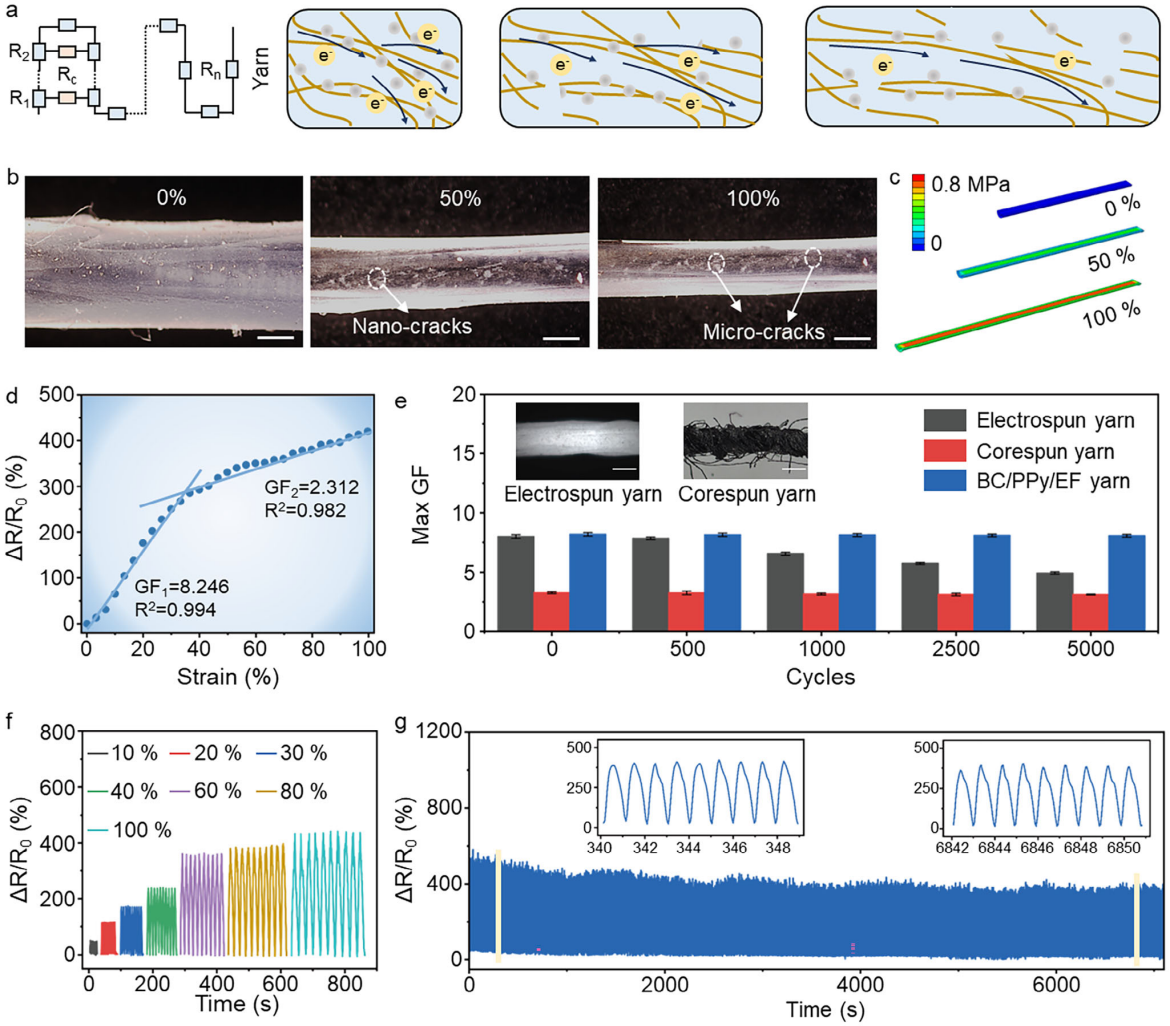
Tunable electrical responsiveness of the yarn strain sensor. (a) The simulative circuit scheme and mechanism of the BC‐based yarn sensor. (b) Optical photographs and (c) finite element analysis (FEA) of the BC‐based yarn sensor during extension, showing cracks and elongation governed by modulus‐mismatch. (d) Relative resistance changes with strain curves. (e) Performance comparison (GF values) between stretchable electrospun yarn, corespun yarn, and BC/PPy/EF yarn sensors during 5000 cycles of 100% stretching. Scale bar 1 mm. (f) Electromechanical response under large‐strain deformation. (g) Repeatability test of BC‐based yarn sensor.

The BC/PPy/EF yarn, especially the conductive BC/PPy core‐layer networks, is highly flexible and stretchable. Upon stretching, the BC/PPy fibers tighten, together with the deformation of the network to dissipate energy. To understand the advantages of yarn design, we compared the sensing mechanisms of film and yarn structures. In the film structure, each PPy‐attached nanofiber can be modeled as an individual resistor (*R*_fiber_) and connected in parallel via contact points between adjacent fibers (Figure S13). The twisted behavior of the yarn substantially reduces interchain distance and modifies the electron transfer (Figure 4a). More adjacent fibers within the network come into contact, transforming the equivalent circuit of the yarn from a purely parallel to a series‐parallel configuration. The total resistance becomes the sum of the on‐state resistances of fibers (*R*_fiber_) and contact resistance (*R*_sc_). We employed optical images to investigate the interface during stretching and finite element analysis (FEA) to simulate the force distribution in the hierarchical yarn (Figure 4b,c). Under tensile loading, twisted PPy/BC fibers tend to orient along the strain direction and generate cracks, extending longitudinally and activating the cracking mechanism [31]. The process evolves through two distinct stages: (i) the appearance of intermittent superficial cracks in the core fibers within 50% strain, followed by (ii) progression to continuous crack networks developing throughout the 3D interwoven architecture within 100% strain. The modulus mismatch, arising from disparities in elastic modulus between core‐sheath components, drives this behavior, characterized by the core's substantially reduced elongation compared to the sheath [32]. Thus, modulus‐mismatch‐governed elongation and cracks potentially might be the key and reason for highly sensitive sensing. Based on the mechanism and results, we considered key material and construction parameters in the design of the yarns. For instance, the sensing networks mixed with conductive 1.5 wt.% PPy is expected to endow yarns with exceptional electrical conductivity, achieving a Δ*R*/*R*_0_ value of ≈400% (Figure S14a). However, the aggregates formed by excess PPy (2.0 wt.%) reduced the initial *R*_0_ of the yarn with an ΔR/R_0_ value of 350%. Different from the direct effect of conductive materials on the electro‐mechanical performance, a trade‐off between strain and *ΔR/R_0_* value needs to be considered, whether the twist of the sensing layer or the thickness of the elastomer. The twist of the conductive film has a direct impact on the looseness of the sensing network and subsequently the stretchable residual space and the spacing between conductive pathways. The degree of twist is positively correlated with the density of the sensing networks, while inversely correlated with the extent of stretchable residual space. Similarly, the capacity to withstand strain improves as the thickness of Ecoflex increases, whereas the sensing sensitivity decreases. Hence, the optimal yarns are achieved by controlling the twist and thickness at 2 T/cm and 1.2 mm (Figure S14b,c).

For our first functional demonstration, the natural integrability and compatibility of yarn make it particularly suitable for smart wearables. According to reports, human epidermis experiences a strain variation of 0%–70%, while for the fine joints of physiological activities (e.g., breathing) becomes much smaller. Therefore, both the sensitivity to low strains and accessibility to large strains are key factors for sensors. As shown in Figure 4d, the sensitivity of yarns could be quantified by the gauge factors (GF), defined as (Δ*R*/*R*_0_). The Δ*R*/*R*_0_ vs strain curve could be fitted as two stages (8.246 and 2.312), representing the sensitivity at low strain levels (below 40%) and large strain levels (40%–100%), respectively. As a comparison, the ordered conductive network distribution of EF‐encapsulated BC/PPy twisted structure realizes enhanced mechanosensitive design and structural stabilization (Figure 4e). Hence, BC/PPy/EF yarn sensor demonstrates the best GF values and robustness performance after comparing with electrospun yarn and corespun yarn sensors. Figure S15 summarizes typical cellulose‐based stretchable resistive sensors, highlighting architecture‐dependent sensing performance [16, 20, 33, 34, 35, 36, 37, 38, 39, 40]. The yarn sensors (yellow area) achieve higher GF values than film sensors (blue area), demonstrating that the 1D yarn configuration provides a substantial enhancement in stretchable resistive sensing. Besides, our twisted BC yarns with a core‐sheath structure exceed that of most yarn sensors, benefiting from the crack‐sensing mechanism. Due to the rapid generation and closure of cracks, the yarn maintains high‐fidelity signal acquisition at subpercentile strain levels and large deformations (Figure 4f; Figure S16a). Since the conductive pathways are only affected by the degree of deformation, independent of frequency, the Δ*R*/*R*_0_ values of the yarn sensor remain constant at different stretch speeds (Figure S16b).

To evaluate the sensor's response characteristics, 50%  stretching was applied to the yarn, and simultaneously recording the starting and ending times of the signal. A rapid response time occurring within 46 ms without electrical hysteresis was calculated in Figure S16c, which is regarded as adequate for monitoring general physiological and joint movements when considering the standard respiration rate of 12–20 breaths per minute and the heart rate of ≈80 beats per minute [41, 42]. Notably, there is a trade‐off between the sensitivity and sensing stability. The yarn demonstrated universal strain overshooting behavior throughout the entire deformation range (20%–100% strain), a direct consequence of resistance hysteresis dynamics governed by Ecoflex's viscoelastic characteristics (Figure S16d) [43, 44]. However, the influence to the sensing stability in the range below 40% strain remains relatively minor owing to the small deformation and high sensitivity. A repeatability experiment was conducted to validate the yarn's stability after repeated stretching, a critical factor for ensuring reliable physiological and motion monitoring (Figure 4g). The low hysteresis yarn sensors warrant consistent and precise signals during 7000 loading‐unloading cycles at 60% strain. The dominant degradation behavior is characterized by the decoupling of the strain responses of the conductive yarns between the core layer and the Ecoflex matrix after exceeding 7000 cycles. To fulfil the unique demands of smart clothes, yarn sensors must adapt to washing and friction conditions, as well as withstand a variety of repeated mechanical stimuli, such as flexion, pressure, and torsion. Figures S17 and S18 demonstrate the constant peak patterns after the mechanical stimulus. The reason could be attributed to the absence of visible crack formation and its disappearance.

### 2.5 Core‐Sheath Yarn for Intelligent Multifunctional Integrated System

**FIGURE 5:**
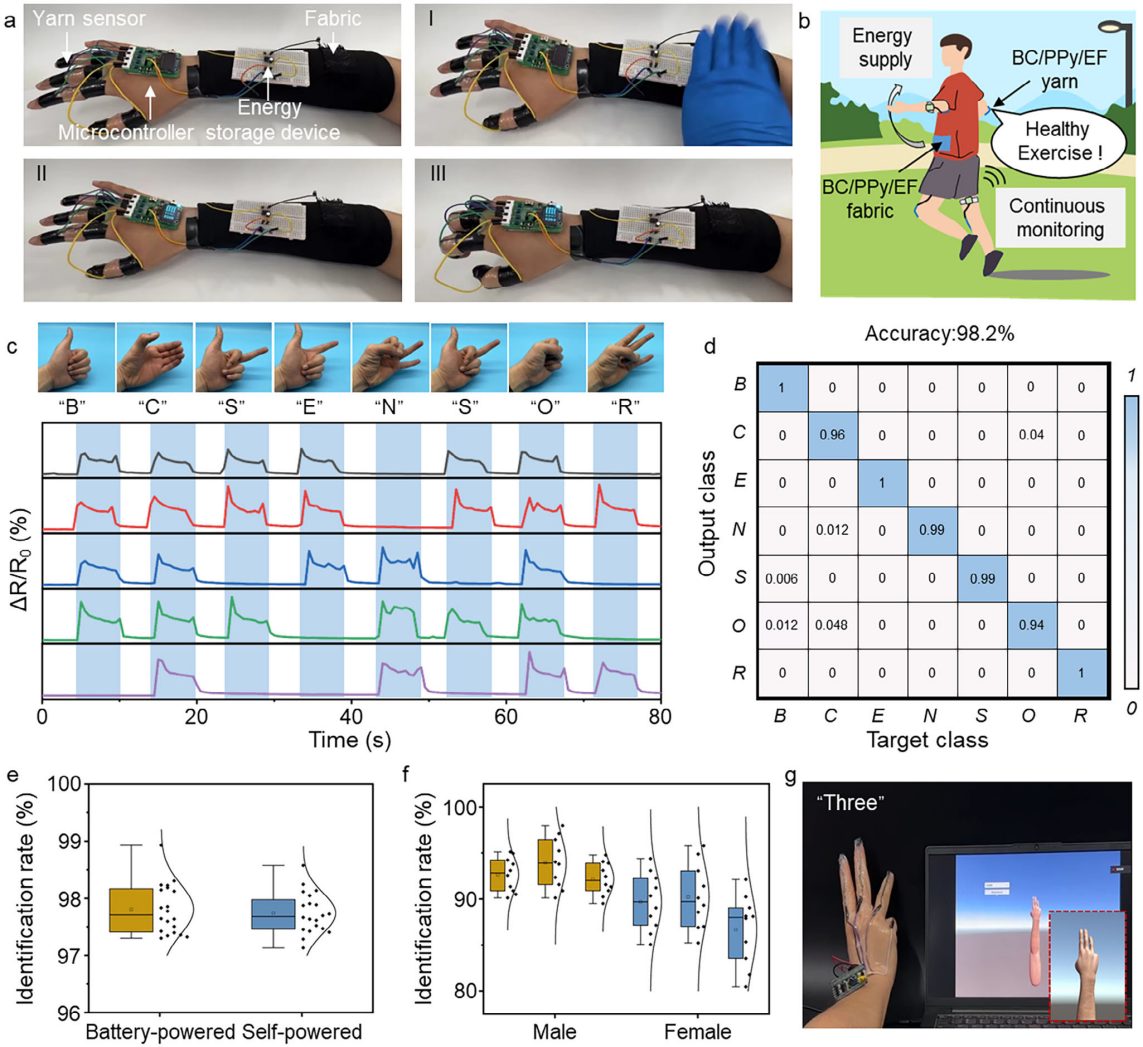
Practical implementation of BC‐based yarn sensor for the integrated energy‐harvesting and strain‐sensing system. (a) The system consists of the energy harvesting fabric, power storage modules, and strain sensing units. (b) Schematic diagram of the healthy monitoring based on the integrated system. (c) Relative resistance changes of language recognition of characters “B,” “C,” “E,” “N,” “S,” “O,” and “R,” (d) Confusion matrix for the testing results. (e) Identification rates among self‐powered and battery‐powered language recognition systems. *n* = 20. (f) Identification rates among different genders. *n* = 10. (g) Applications of BC‐based yarn sensor for VR interaction.

Given the mechanosensitive twisting structure and modulus‐mismatch behavior of core‐sheath structured sensors, which demonstrate outstanding sensibility and natural conformability, the BC/PPy/EF yarn holds great potential allowing physiological and movement monitoring (Figure S19). System functionality was demonstrated through comprehensive motion analysis, encompassing both physiological signatures (vocal articulation and respiratory rhythms) and kinematically significant movements (cranio‐cervical motion, humeral flexion, and locomotion) that collectively model athletic biomechanics. As an illustrative validation, the above‐mentioned feature inspires us to design a multifunctional platform by integrating the yarn‐based strain sensor with a fabric‐based triboelectric nanogenerator, enabling simultaneous self‐powering and human health monitoring. Moreover, the microcontroller also acts as the signal acquisition and communication device that contains the Micro Control Unit (MCU), signal acquisition circuits, and a Bluetooth module. Electrical signals provided by the microcontroller were then transmitted to the host computer for signal identification and image processing (Figure S20). We employed an energy harvesting device to capture and store the electrical charge generated by mechanical deformation of the fabric, subsequently powering the yarn via a microcontroller (Figure 5a; Movie S1). When fully charged, the capacitor provides power for healthy monitoring systems, completing a multifunctional integrated energy harvesting and consumption system (Figure 5b). The yarn sensor established a real‐time sign language decoding system via the distributed piezoresistive yarn networks mounted on five joints, innovative self‐powered energy harvesting technology, and parallel‐configured electronic interfaces enabling multiplexed sign language recognition and analysis (Figure 5c). Prior to testing, we established standardized letter‐to‐gesture correspondences and synchronized signal acquisition protocols during dynamic joint articulation. Machine learning algorithms subsequently correlated characteristic resistance patterns with target letters (B, C, S, E, N, O, R). Since there are obvious differences in the signals generated by bending and stretching of fingers, digital gestures can be distinguished in real‐time by the resistance signals of five fingers. For instance, when configuring the hand gesture for the letter “S” with an extended thumb and curved four fingers, the corresponding yarn sensors on the four fingers showed a marked increase in Δ*R*/*R*_0_ signals, while no Δ*R*/*R*_0_ values were collected for the thumb. When we mix 200 grasping data for the foregoing letters together and randomly select 80% data for training, reserving the additional 20% data for recognition. The recognition accuracy after 1000 iterations of training reached approximately 98.2% in identifying the letters (Figure 5d). The visualizations in the t‐SNE clustering with legend demonstrated that the distinct character patterns are readily distinguishable (Figure S21). Notably, the majority of incorrect recognitions are for the letters “O” and “C,” which are attributed to the similarities in gestures and require more analysis regarding peak intensity.

As shown in Figure 5e, the self‐powered sign language recognition system demonstrated similar recognition accuracy when compared to the battery‐powered system, showing the practicality and applicability of multifunctional integration of strain sensing and energy self‐sufficiency. The users' intended expression, regardless of gender, can be discerned by the data diagram according to the knuckles stretching and bending (Figure 5f). Although weak signal interference is presented in real‐time gesture recognition, the reliable accuracy still enables an in‐depth human‐machine interface for potential VR‐game and VR‐exercise (Figure 5g; Movie S2). Furthermore, the system was integrated into a plantar pressure distribution system for yoga training monitoring (Figure S22). The voltage output signals and the corresponding pressure maps can reflect the plantar pressure distribution, thereby enabling the monitoring of yoga exercises. We anticipate that further advancing the design of these multimodal sensor arrays, regulating the permutation distribution, and integrating them with other materials will allow more accurate and informative data to be collected and analyzed.

## 3 Conclusion

In summary, the stretchable flexible electronic yarns with ordered conductive cellulose networks and core‐sheath structures provide an integrated multifunctional platform with unprecedented strain sensing and self‐powering. Based on the yarn structure, a triboelectric yarn aggregation was developed to not only realize energy harvesting (over 40 V) but also power the wearable devices integrated with yarn sensors for continued operation. The twisting behavior and core‐sheath design effectively enhance the orientation of conductive BC networks and stretchability of 1D linear structure, thereby influencing the contact manner of sensing fibers and crack deformation mechanism. The yarn sensor gives a high mechanosensitive response (8.246), a wide strain range (up to 100%), and long‐term reproducibility (7000 cycles). With facile, small‐period, and scalable in situ microbial engineering and textile technology, the proposed yarn sensor will promote the development of next‐generation all‐fiber‐based wearable electronics with a functional integration of strain sensing and energy self‐sufficiency.

## Author Contributions

C.G., D.X., H.S., P.S., Z.G., Z.Z., and K.L. conceived and planned this research. C.G., D.X., H.S., P.S., Z.G. performed the experiments. D.X. performed the mechanical modeling analysis. C.G., K.L. H.S., P.S., Z.G., and Z.Z. organized the data and wrote the manuscript. All authors discussed the results and approved the final version of the manuscript.

## Conflicts of Interest

The authors declare no conflict of interest.

## Supporting information




**Supporting File 1**: advs74514‐sup‐0001‐SuppMat.docx.

**Supporting File 2**: advs74514‐sup‐0002‐MovieS1.mp4.

**Supporting File 3**: advs74514‐sup‐0003‐MovieS2.mp4.

## Data Availability

The data that support the findings of this study are available from the corresponding author upon reasonable request.
