# Supplementary material for: Highly Aligned Bacteria Cellulose Yarn Aggregation for Energy Generation and Strain Sensing
Source: Adv Sci (Weinh). 2026 Mar 19;13(25):e23263. doi: 10.1002/advs.202523263 (PMC13137808; doi:10.1002/advs.202523263)
Supplement: Supplementary file 1 — Supporting File 1: advs74514‐sup‐0001‐SuppMat.docx. [file ADVS-13-e23263-s003.docx]

Supporting Information

**Highly Aligned Bacteria Cellulose Yarn Aggregation for Energy Generation and Strain Sensing**

Chong Gao, Duo Xu*, Hui Sun, Sun Ping, Zongxue Gu, Keshuai Liu, Zhiqiang Zhou*, Bin Yu*, Jian Fang*, and Weilin Xu

C. Gao, H. Sun, Z. Zhou, B. Yu

College of Textile Science and Engineering

Zhejiang Sci-Tech University

Hangzhou 310018, P R China

E-mail: zhouzq@zstu.edu.cn (Z. Zhou), [yubin7712@zstu.edu.cn](mailto:yubin7712@zstu.edu.cn) (B. Yu)

C. Gao, D. Xu, P. Sun, Z. Gu, K. Liu, W. Xu

Stake Key Laboratory of New Textile Materials and Advanced Processing

Wuhan Textile University

Wuhan 430200, P R China

E-mail: [dxu@wtu.edu.cn](mailto:dxu@wtu.edu.cn) (D. Xu)

J. Fang

College of Textile and Clothing Engineering

Soochow University

Suzhou 215123, P. R. China

E-mail: [jian.fang@suda.edu.cn](mailto:jian.fang@suda.edu.cn) (J. Fang)

**This PDF file includes:**

Supplementary Method S1.

Figure S1 to S19.

Movie S1. Wearable closed-loop system integrated with yarn sensors and self-powered fabric for energy generation and strain sensing functions.

Movie S2. Wearable sensing system of our yarns for in-depth human-machine interface.

Experimental Section

*Materials and Chemicals:* Polypyrrole (PPy) was purchased from Sigma-Aldrich., China. Glucose, peptone, yeast, dibasic sodium phosphate (Na_2_HPO_4_‧12H_2_O), citric acid, and sodium hydroxide were provided by Sinopharm Chemical Reagent Beijing Co., Ltd., China. All reagents were of analytical grade without further purification. *Gluconacetobacter xylinus* (strain 1.1812, 3.8 × 10^5^ cfu mL^−1^) was provided by the Institute of Microbiology, Chinese Academy of Science.

*Fabrication of the BC/PPy/EF Yarn:* The yarn was prepared a four-step process: (i) In situ fermentation process to produce the conductive BC/PPy films; (ii) Laser micromachining was employed to shred into wet, rough, conductive shreds with controlled dimensional uniformity; (iii) A wet-twisting process to construct the BC/PPy yarn; (iv) Encapsulating the BC/PPy yarn with Ecoflex. Steps 1 and 4 were performed according to our previously reported methodology^[1-3]^. Basically, various concentrations of PPy (0.1 wt% to 0.4 wt%) were employed as conductive materials to fabricate the BC/PPy films. The bacterial medium was subsequently transferred to an autoclave (LDZX-50KBS, Shanghai Shenan, China) and heated to 120 °C for 30 min. After a 5-7 days′ cultivating period, the BC/PPy films were obtained for further steps. A uniform interpattern pitch of 10 mm was maintained throughout laser micromachining.

*Characterization:* The morphologies and surface structures were characterized using SEM (Sigma 300, Zeiss, Germany) and digital microscopy (DSX510, Olympus, Japan). The chemical composition and surface functional groups were analyzed using FT-IR spectroscopy (Nicolet 6700, Thermo Fisher, USA), XPS (K-Alpha, Thermo Scientific, USA) with an Al Kα radiation source, and XRD (SmartLab SE, Rigaku, Japan) with Cu Kα radiation in the range of  5° to 90°. The mechanical performance was examined using an electronic tensile testing machine (INSTRON 5943, Instron, USA). The cellulase solution was prepared by mixing 60 mL of acetic acid-sodium acetate buffer with 0.3 g of cellulase. The BC/PPy yarn was immersed in the enzyme solution at a temperature of 50 °C. To investigate the sensing performance, a flexible electronic test system provided by Shanghai Shentai Intelligent Technology Co., Ltd., China was used to apply force, and a TruEbox 02CM system (LinkZill, China) was used to record the electric current (*I*). The output voltage signals generated by the yarn fabric was collected using a linear mechanical motor (LinMot E1100) and an electrostatic meter (Keithley 6514, USA). The electric potential transformation of the fabric during contacting and separating states is simulated using COMSOL multiphysics. The orientation structures of control and Ecoflex encapsulated yarns were conducted by wide-angle X-ray scattering (WAXS), which was carried out by a Xeuss 2.0 system (Xenocs SA, France) equipped with Cu Kα X-ray source (λ = 0.154189 nm) at 50 kV and 0.6 mA.

**
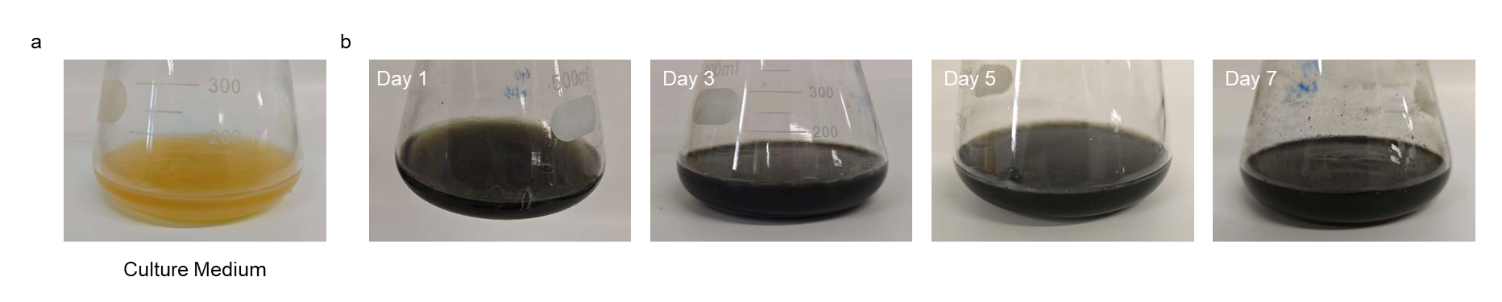
**

Figure S1. Optical photographs of the cultivating process. Culture medium of (a) BC without functional materials, and (b) BC/PPy.


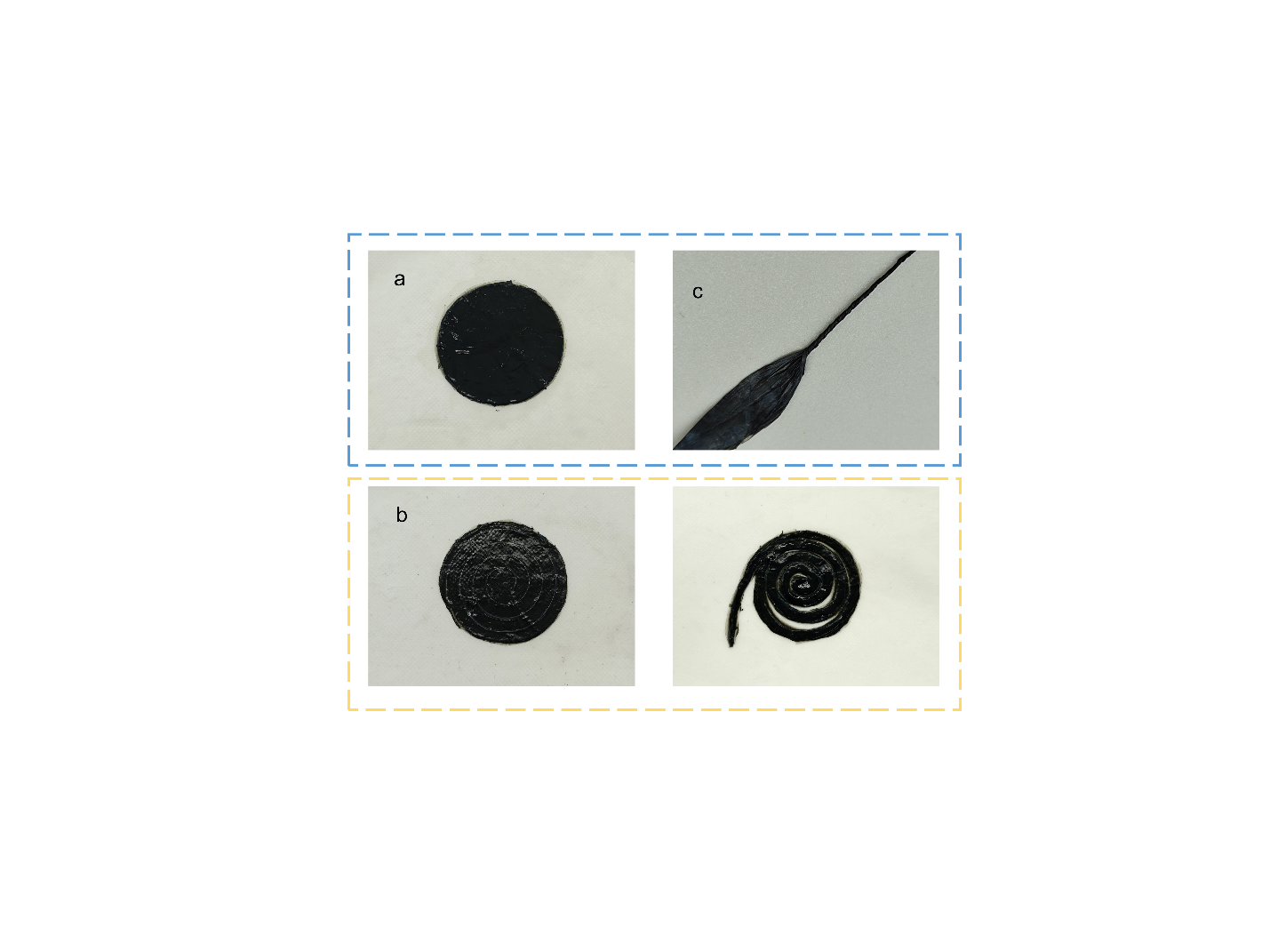


Figure S2. Photographs of the BC/PPy (a) film, (b) rough shred, and (c) yarn.


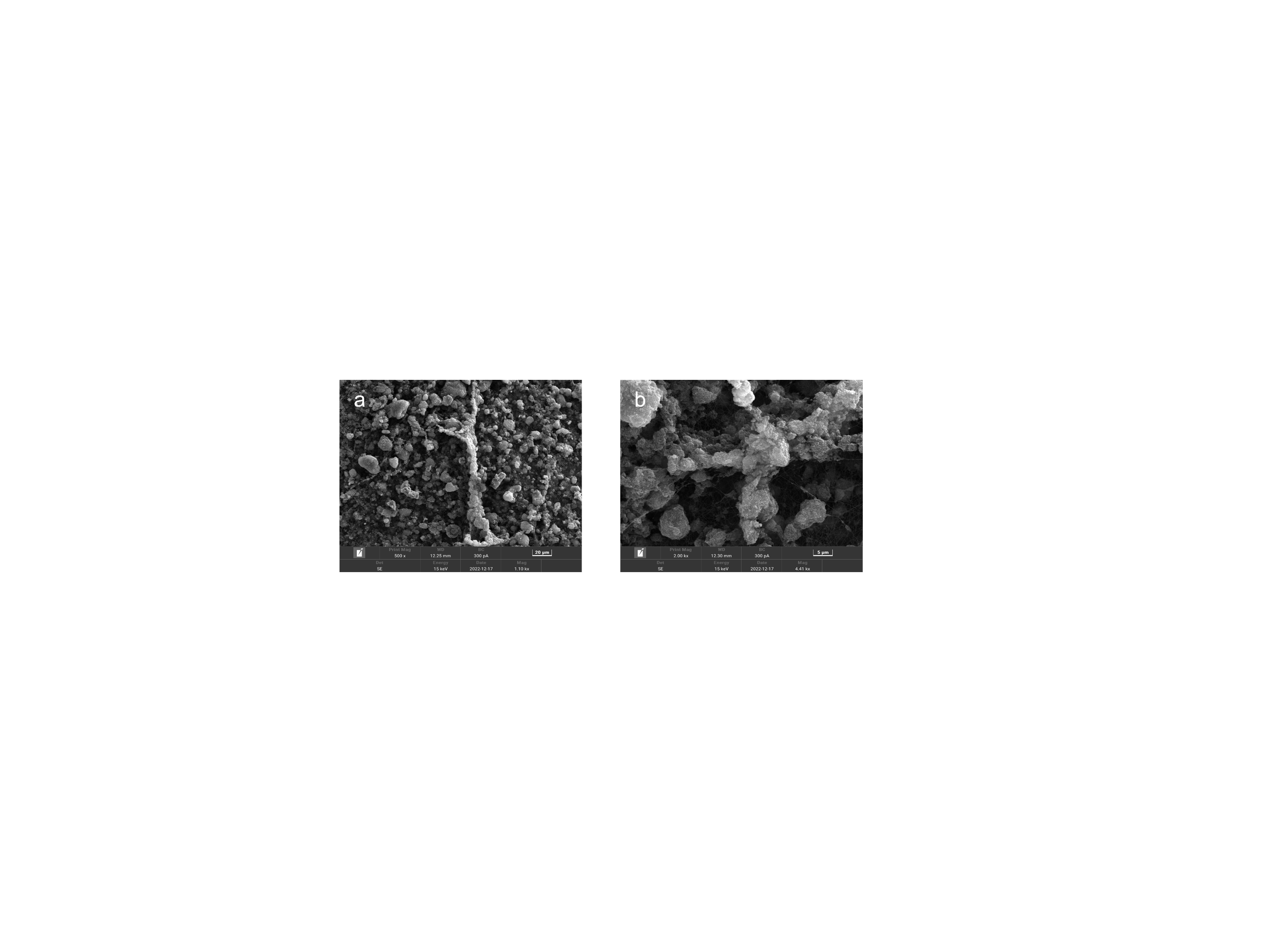


Figure S3. The interconnected networks of conductive fibers.


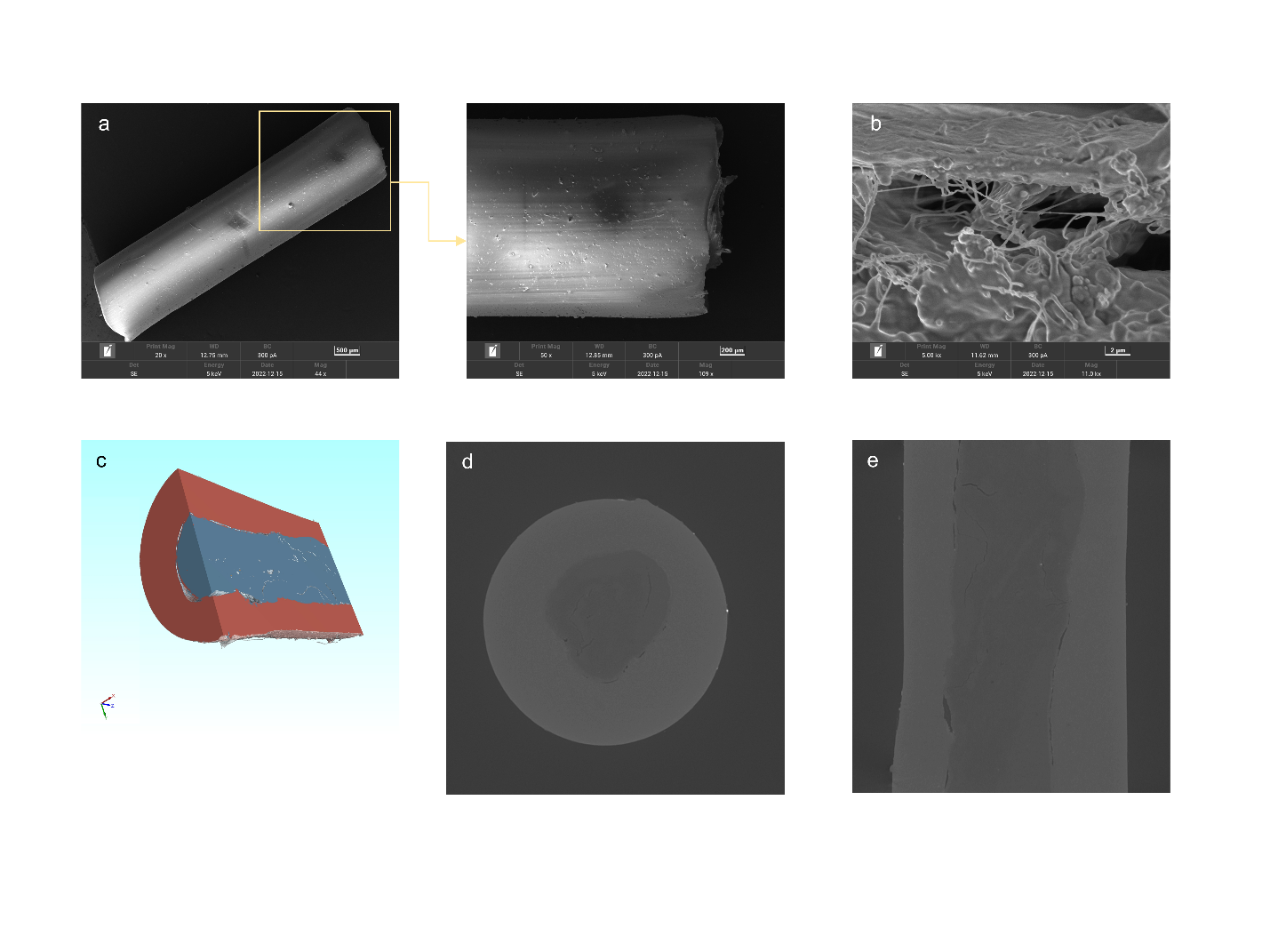


Figure S4. The (a) surface morphologies of BC/PPy/EF yarn and (b) adhesion state of BC/PPy. (c-e) The micro-CT images of BC/PPy/EF yarn.


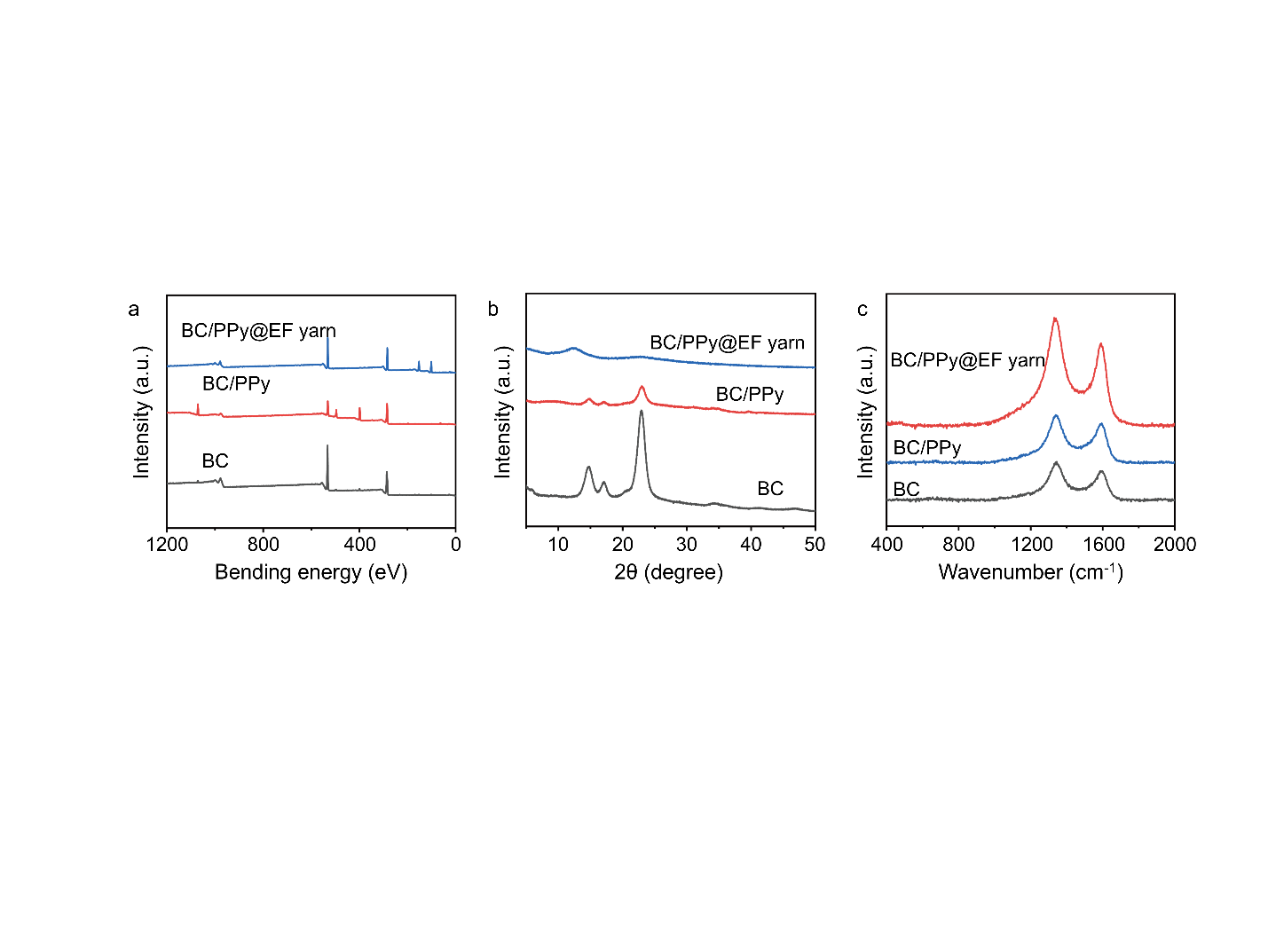


Figure S5. (a) FTIR spectra, (b) XRD and (c) XRS patterns of BC, BC/PPy, and BC/PPy/EF yarn.


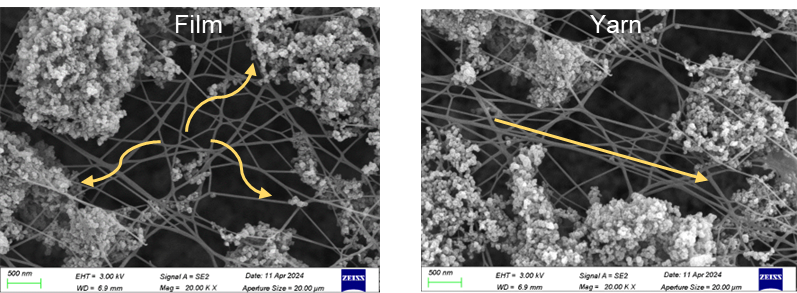


Figure S6. SEM images show random BC/PPy fibers become more aligned after twisting.


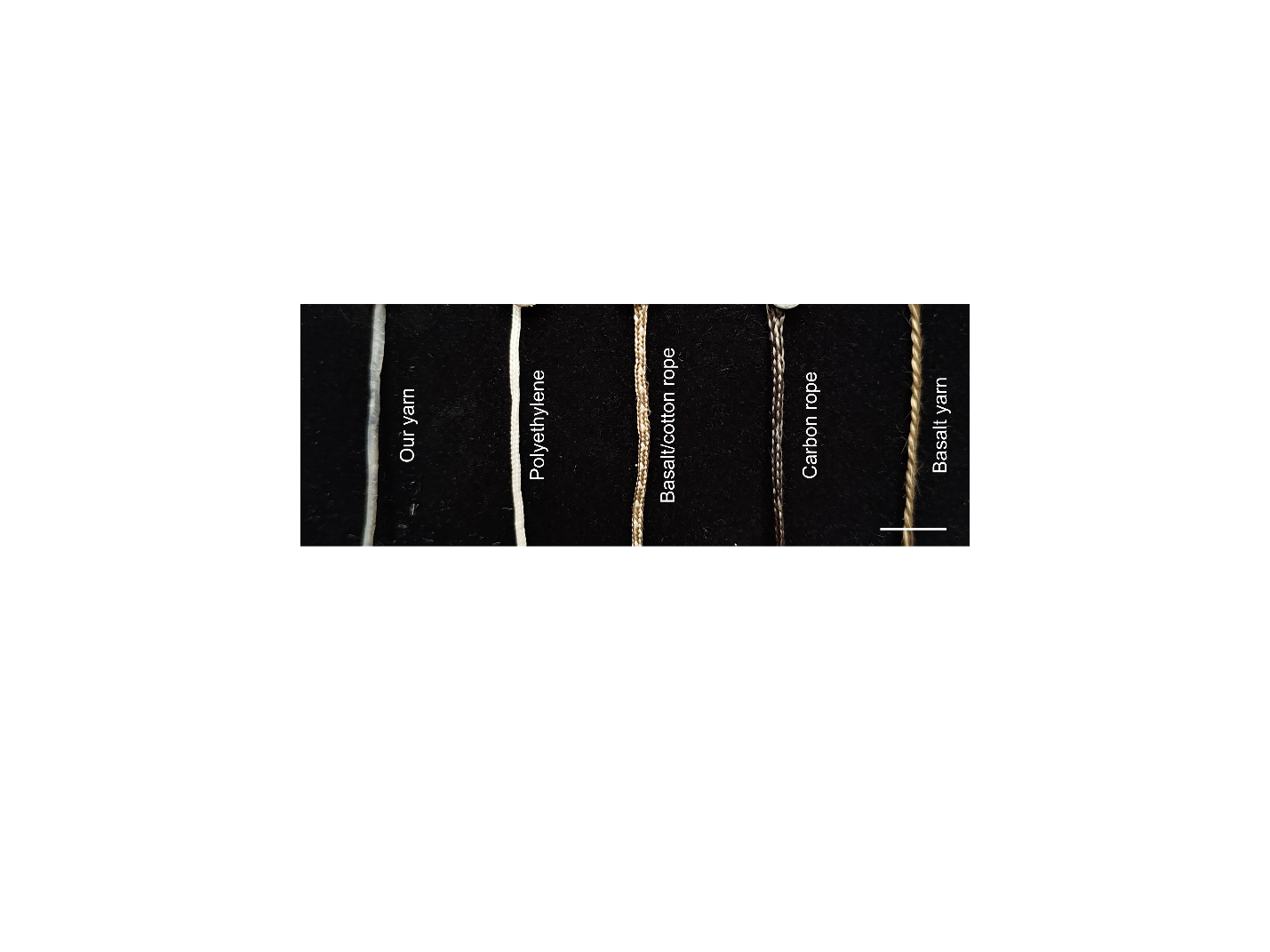


Figure S7. Comparation of thickness of comment yarns and ropes with our yarn.


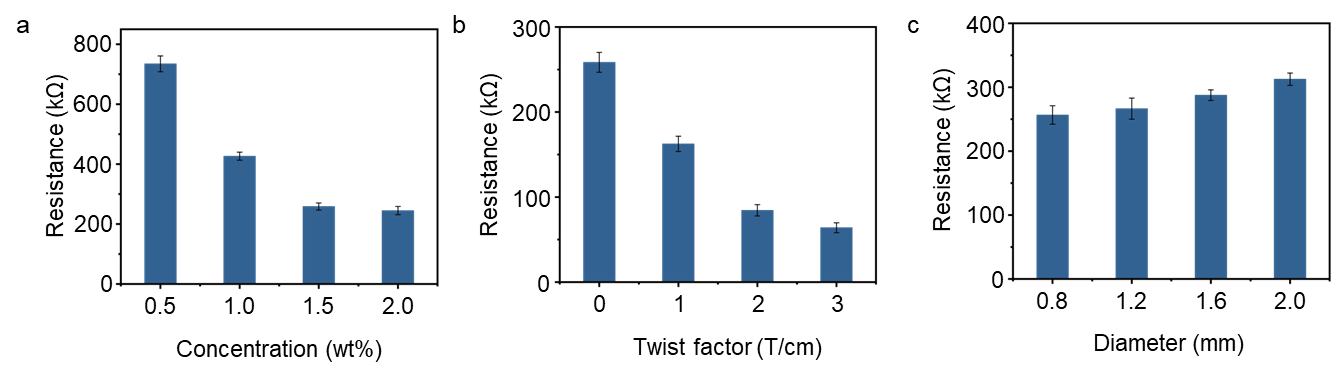


Figure S8. Resistance of BC/PPy/EF yarns under various (a) PPy concentration, (b) twist degree, and (c) yarn thickness.


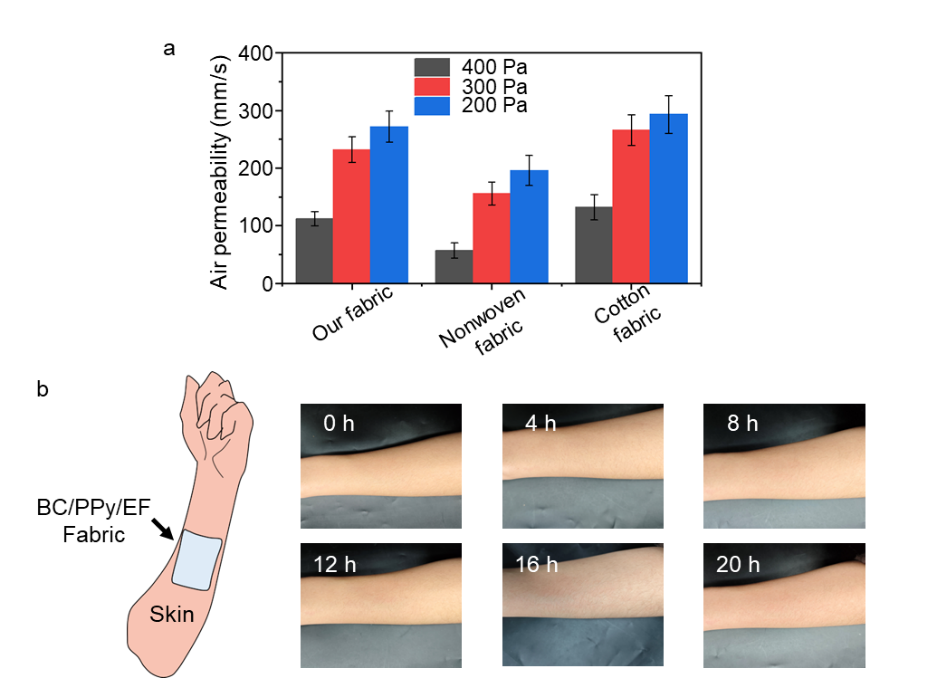


Figure S9. (a) Air permeability evolution of cotton fabric, nonwoven fabric, and our fabric under different pressure (200, 300, 400 Pa). (b) Skin-interface properties of a BC/PPy/EF fabric.


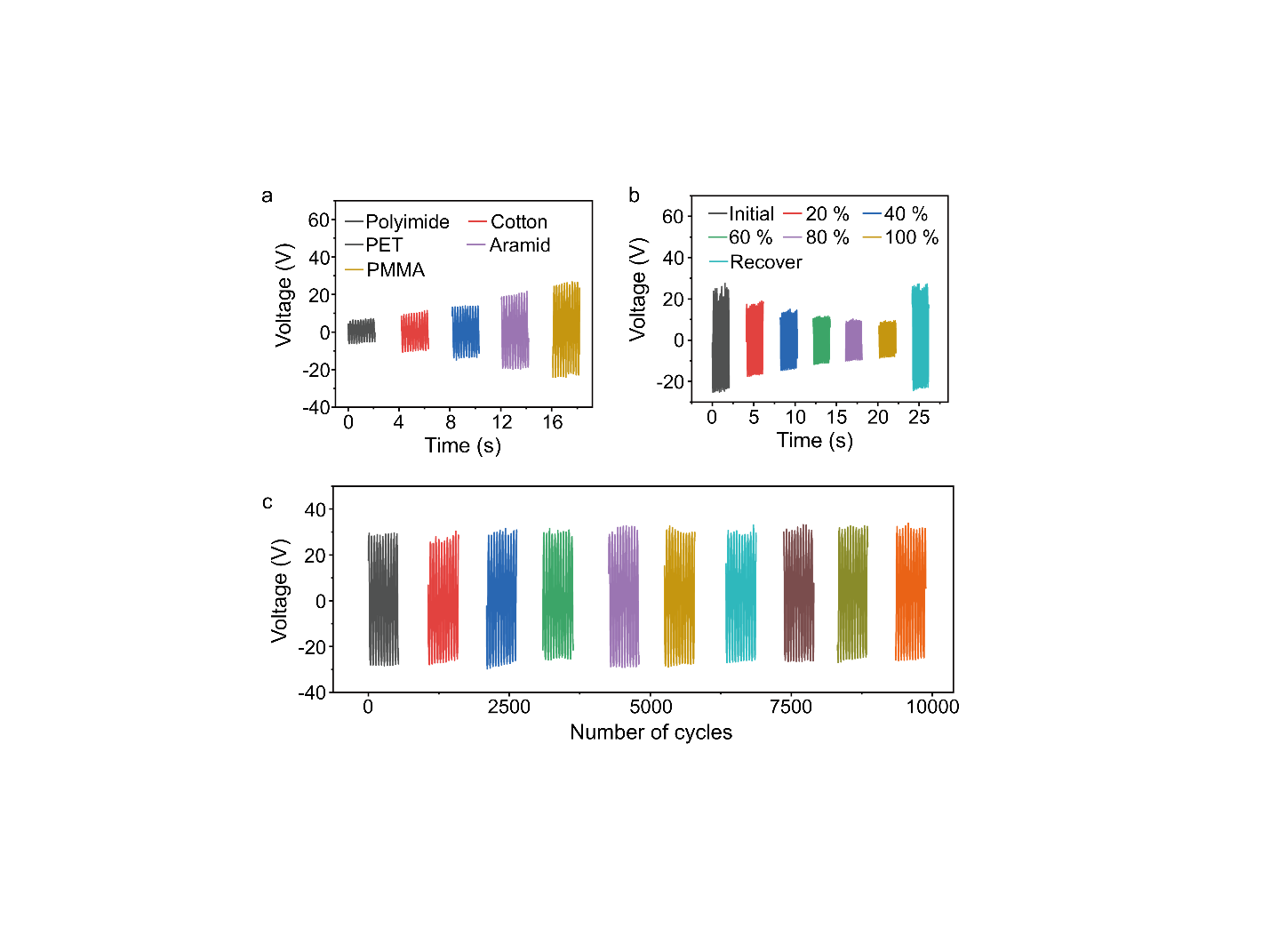


Figure S10. Output voltage signals of the yarn fabric. Voltage output (a) when sensing different positive triboelectric layer materials and (b) under different stretching strain. (c) Durability evolution of the yarn fabric for 10000 cycles.


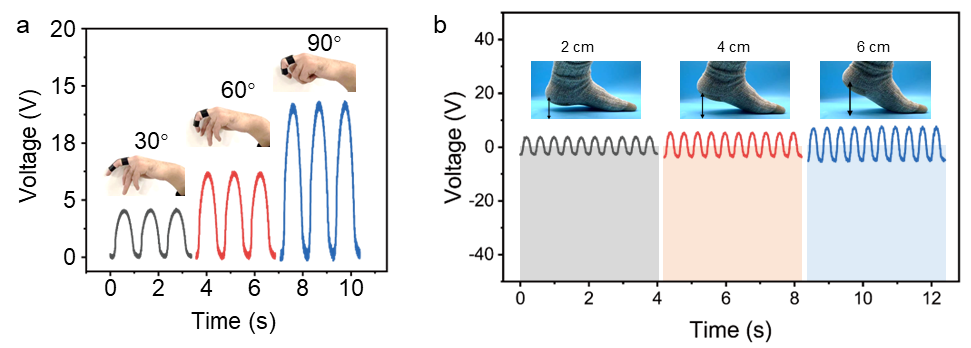


Figure S11. Energy generation behaviors when (a) bending fingers, and (b) pedaling with different height.


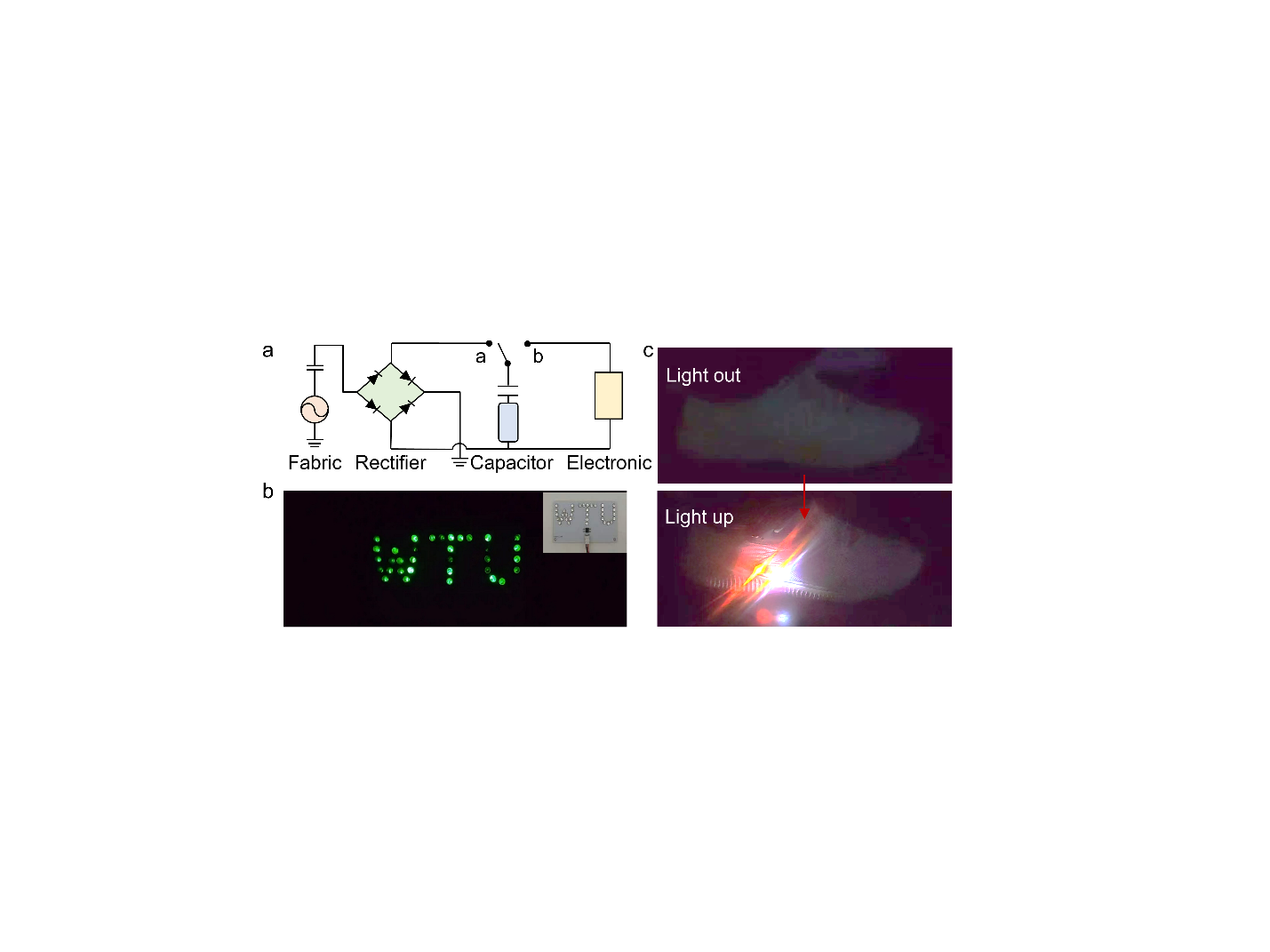


Figure S12. Applications of the yarn fabric in self-powered functions. (a) The designed equivalent circuit for driving electronic devices. Application of the yarn fabric for lighting up the (b) “WTU” LED arrays induced by hand tapping, and (c) the integration on shoes by walking.


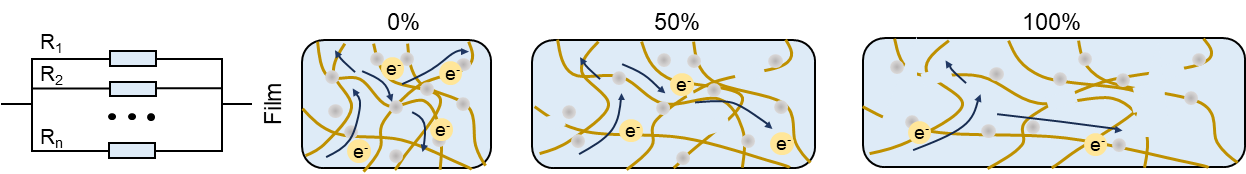


Figure S13. The simulative circuit scheme and mechanism of the BC-based film sensor.


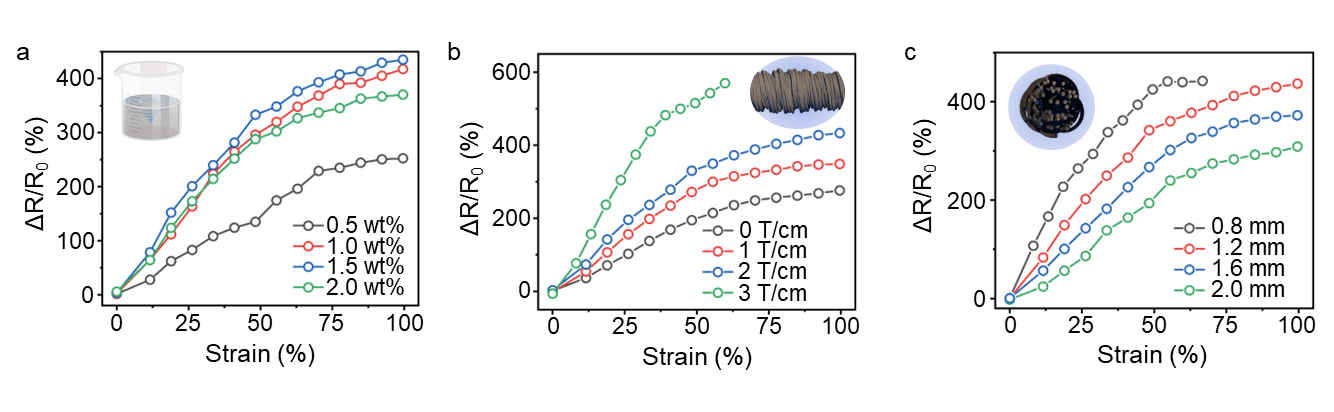


Figure S14. Δ*R/R*_0_ values of BC/PPy/EF yarns under various (a) PPy concentration, (b) twist degree, and (c) yarn thickness.


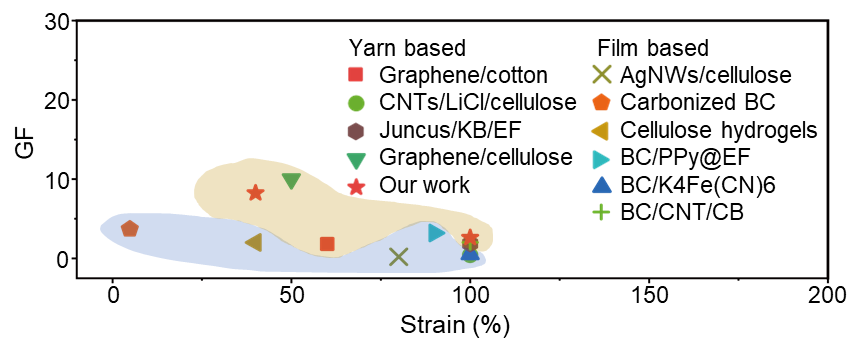


Figure S15. Performance comparison (GF values and strain working ranges) of existing stretchable cellulose-based resistive sensors in film and yarn architectures.


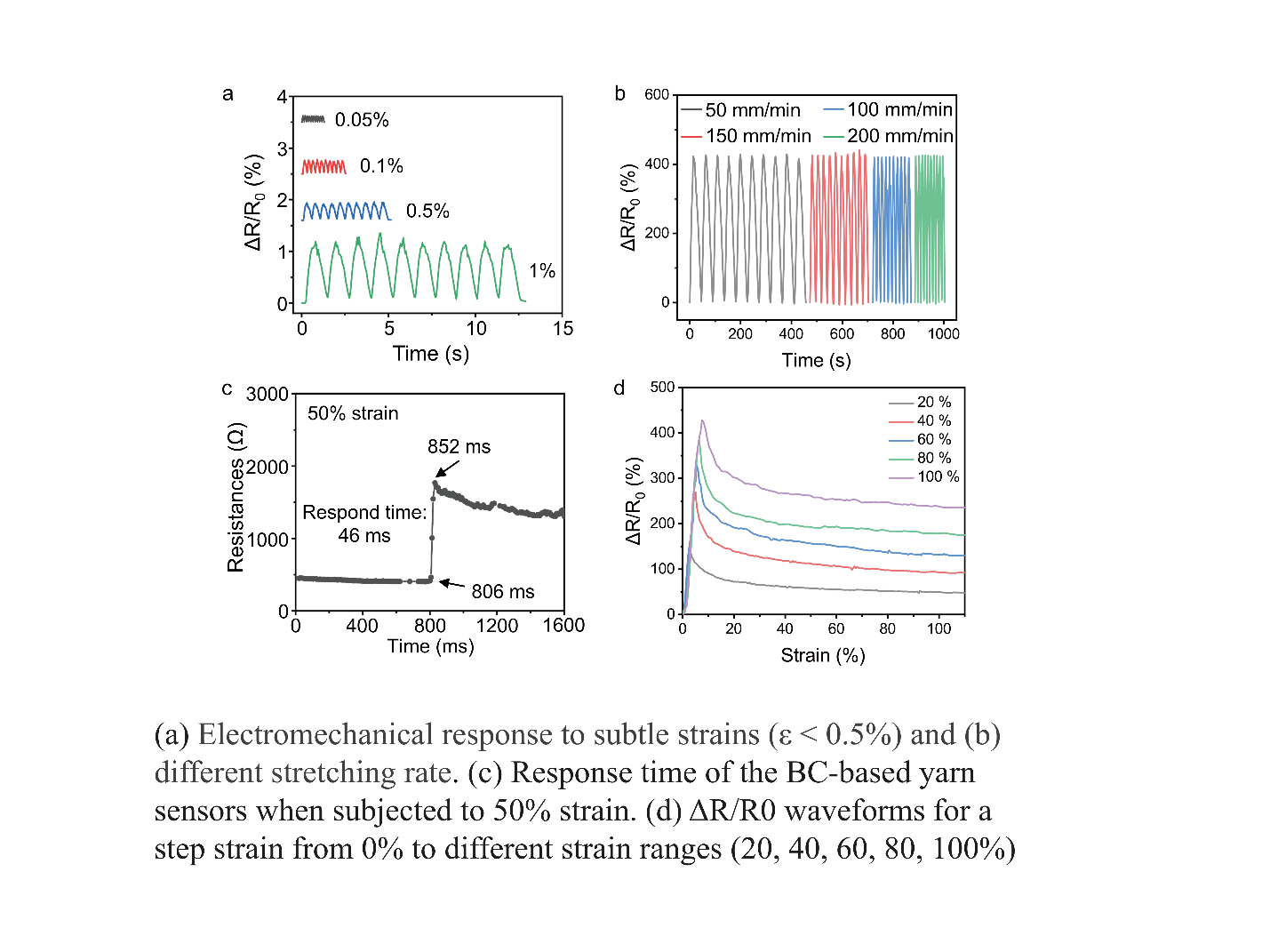


Figure S16. Sensing performance of BC/PPy/EF yarn. (a) Electromechanical response to subtle strains (ε < 0.5%) and (b) different stretching rate. (c) Response time when subjected to 50% strain. (d) *ΔR/R_0_* waveforms for a step strain from 0 to different strain ranges (20, 40, 60, 80, 100%).


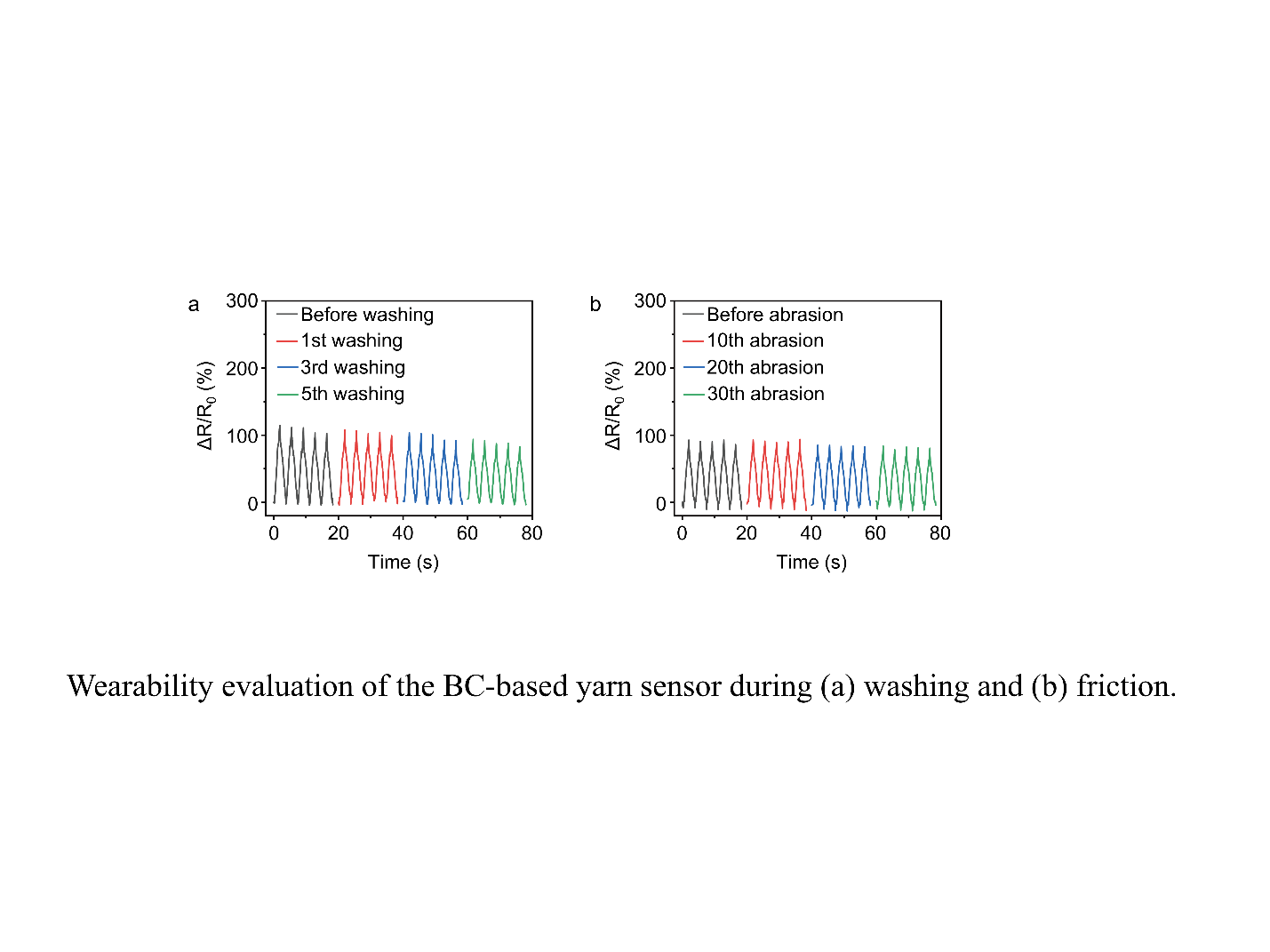


Figure S17. Wearability evaluation of the yarn sensor during (a) washing and (b) friction.


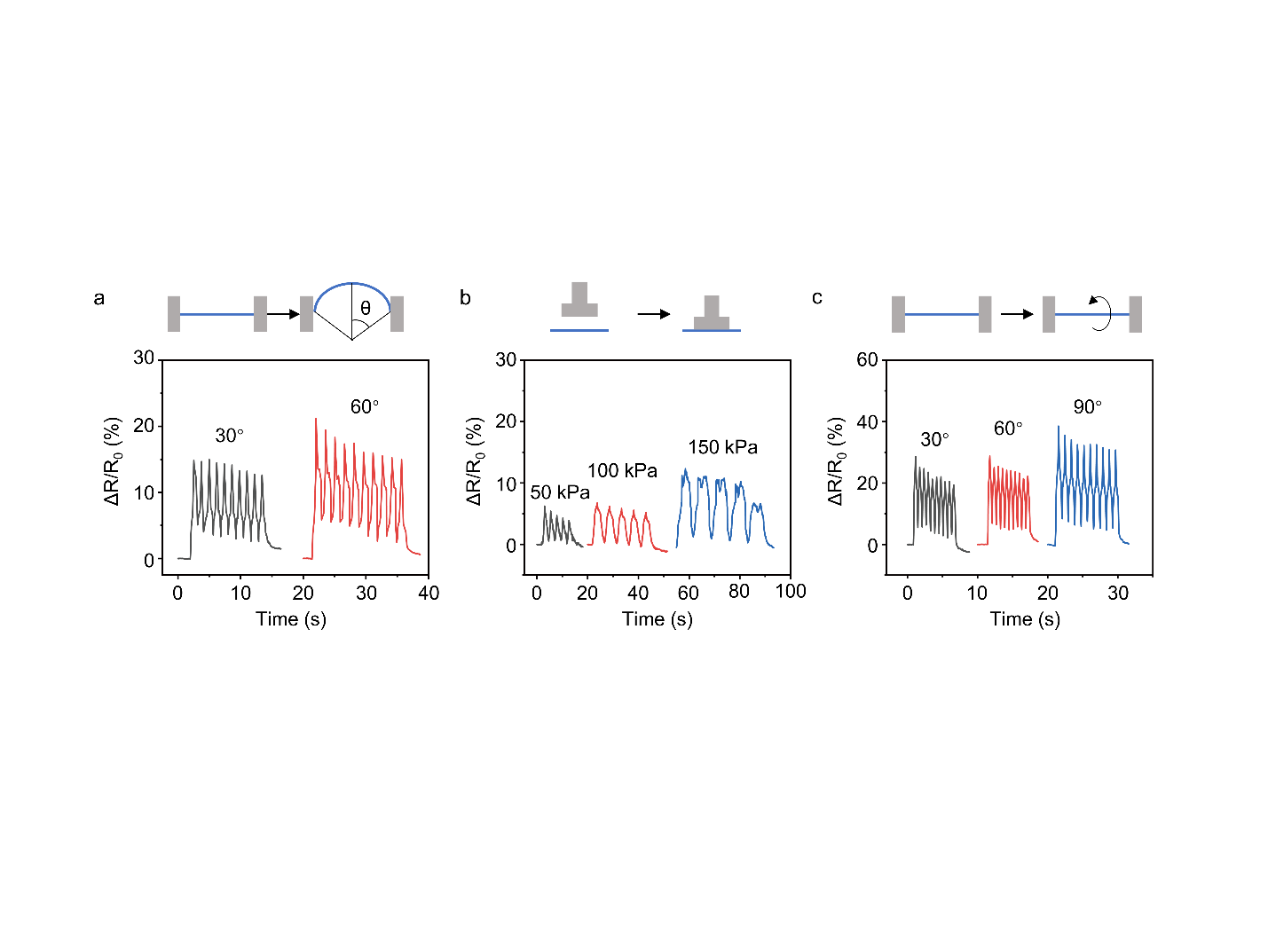


Figure S18. Sensing performance of the yarn sensor under repeated mechanical stimuli in practical, such as flexion, pressure, and torsion.


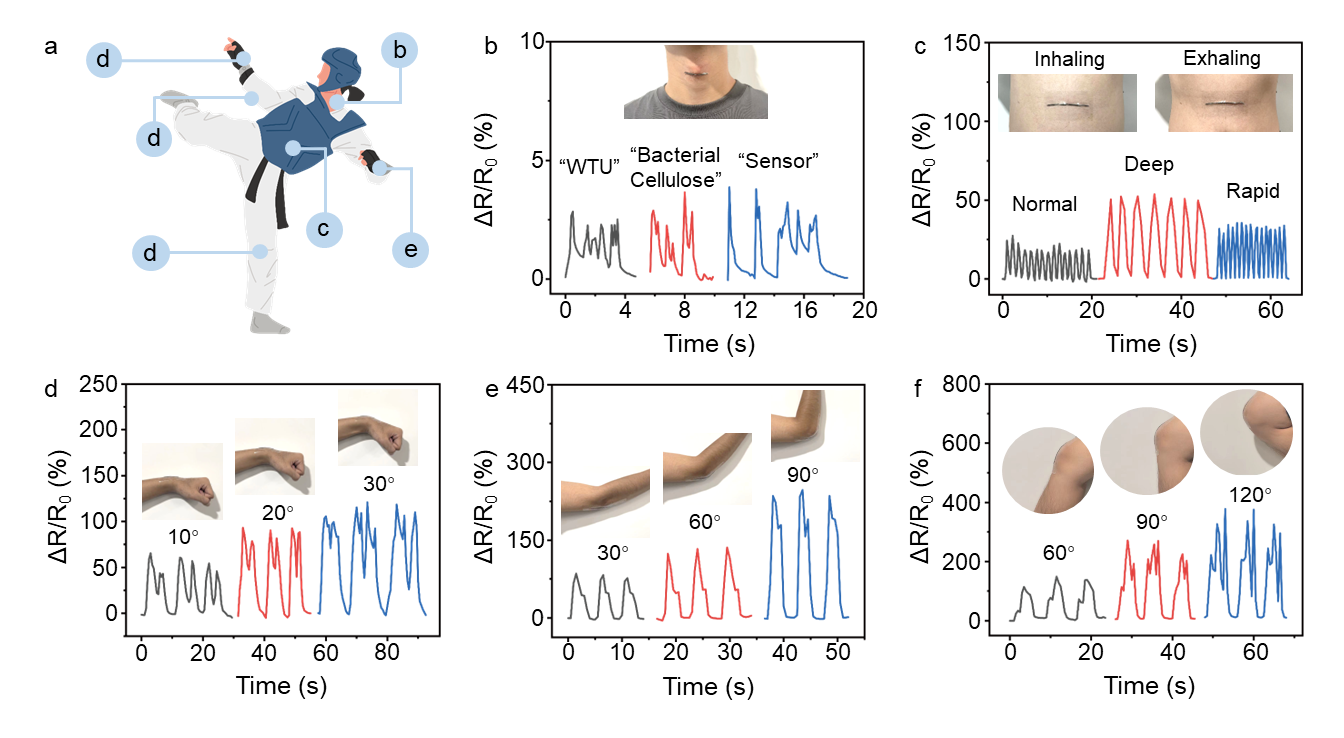


Figure S19. BC/PPy/EF yarn sensor for human monitoring. (a) Wearable yarn sensor deployment schematic on body regions. Output relative resistive waveforms recorded for distinct motion exercises such as (b) voice recognition, (c) respiration, typical joint movement: (d) wrist, (e) elbow, (f) knee, and (g) sign language recognition. As shown in Fig. S18b, when reading “WTU”, “Bacterial Cellulose” and “Sensor”, the ΔR/R0 curves presented unique patterns with under 5%. This result demonstrates the remarkable sensitivity of the cellulose yarn. Compared to vocalization, the abdominal offset in breathing exhibits a greater magnitude, tested to approaching ∆R/R0 of 50%, making it more suitable to evaluate physical functions (Fig. S18c). Multiple respiratory states were examined, in which deep breathing generated signals characterized by higher peak values and lower frequency than normal breathing, whereas frequency markedly elevated when breathing rapidly. The surveillance of human motion rate and peak intensity becomes more crucial for health management. Figs. S18d-f respectively shows the ΔR/R0 signals produced by the wrist, elbow, and kneecap when bending at a certain angle. It is evident that the greater the strain generated by the joints, the higher the level of the relative electrical signals. These subject area covers a large spectrum of motion and physiological information and offer a broad ∆R/R0 signal from approximately 5% to 400%, which may serve as representative signals of the primary strain stimuli on the human body.

*
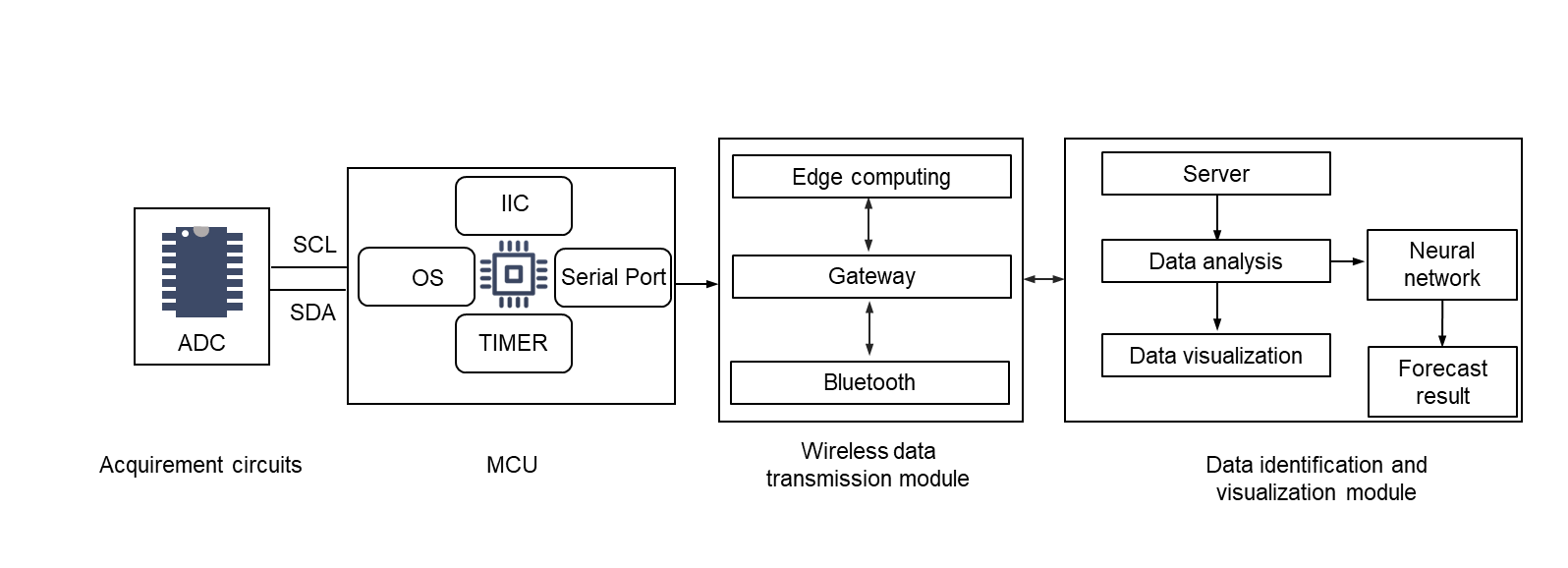
*

**Figure S20. Schematic diagram of the data acquirement and visualization modules of the posture monitoring system.**


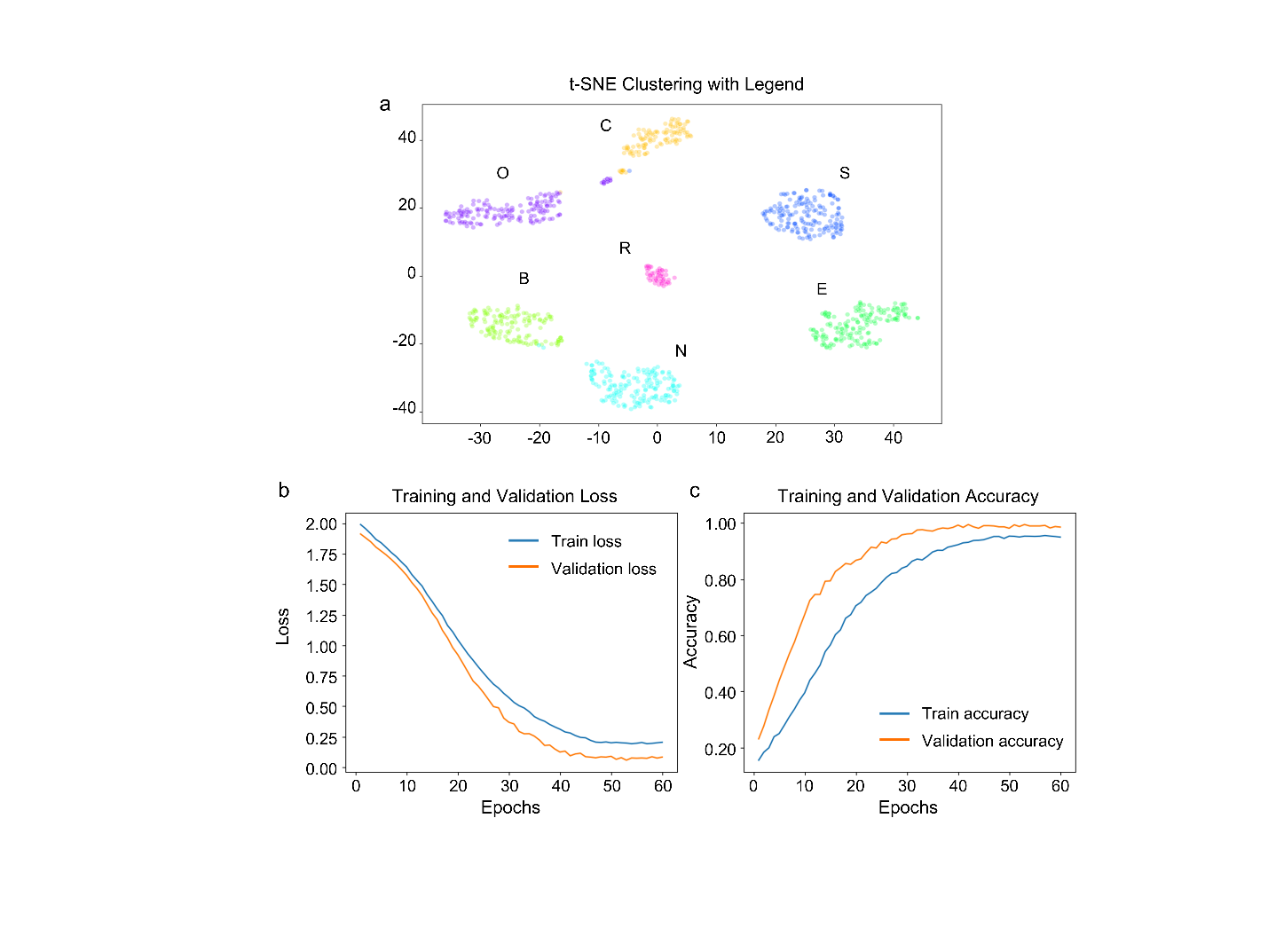


Figure S21. Visualization analysis and prediction of personal gait data. (a) The t-SNE visualization of the clustered data of different character. The variation in (b) loss and (c) accuracy during training with increasing epochs.

**
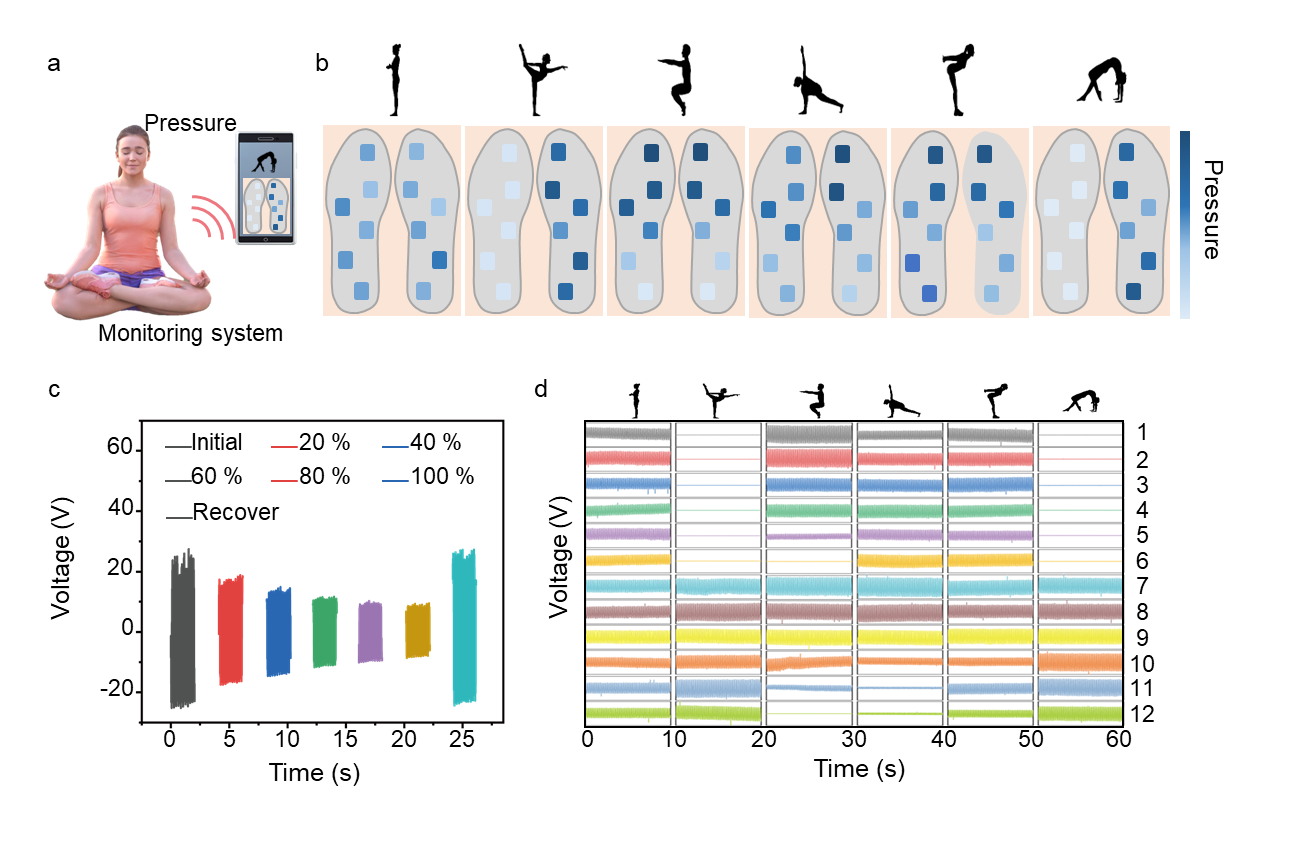
**

Figure S22. The integrated energy-harvesting and strain-sensing system for healthy monitoring in yoga exercise. (a) Schematic diagram of the energy-harvesting and monitoring during wear. (b) The plantar pressure maps demonstrate the motion pressure of a volunteer wearing the integrated platform. (c) The generated voltage output and the corresponding strain during wear. (d) Voltage output signals of six typical poses in yoga.

Reference

1. C. Gao, Y. Liu, F. Gu, Z. Chen, Z. Su, H. Du, D. Xu, K. Liu, W. Xu, Biodegradable Ecoffex encapsulated bacterial cellulose/polypyrrole strain sensor detects motion with high sensitivity, ffexibility and scalability, *Chem. Eng. J.* 460, 141769 (2023).

2. C. Gao, Y. Liu, Z. Gu, J. Li, Y. Sun, W. Li· K. Liu, D. Xu, B. Yu, W. Xu, Hierarchical Structured Fabrics with Enhanced Pressure Sensing Performance Based on Orientated Growth of Functional Bacterial Cellulose, *Adv. Fiber Mater*. 6, 1554–1568 (2024).

3. J. Li, Y. Liu, Z. Gu, P. Sun, K. Liu, D. Xu, C. Gao, W. Xu, Scalable, Green, Flexible Photochromic Bacterial Cellulose for Multicolor Switching, Photo-patterning, and Daily Sunlight UV Monitoring, *Small*, 20, 2309514 (2024).
